# Supplementary material for: Homoeologous non-reciprocal translocation explains a major QTL for seed lignin content in oilseed rape (Brassica napus L.)
Source: Theor Appl Genet. 2023 Jul 13;136(8):172. doi: 10.1007/s00122-023-04407-w (PMC10345078; doi:10.1007/s00122-023-04407-w)
Supplement: Supplementary file 1 — Supplementary file1 (PDF 223 kb) [file 122_2023_4407_MOESM1_ESM.pdf]

Homoeologous non-reciprocal translocation explains a major QTL for seed lignin content in oilseed rape (*Brassica napus* L.)

Hanna Marie Schilbert[1,2,†], Karin Holzenkamp[3,†], Prisca Viehöver[1], Daniela Holtgräwe[1], Christian Möllers[3]

[1] Genetics and Genomics of Plants, CeBiTec and Faculty of Biology, Bielefeld University, Bielefeld, Germany
[2] Graduate School DILS, Bielefeld Institute for Bioinformatics Infrastructure (BIBI), Faculty of Technology, Bielefeld University, Germany
[3] Department of Crop Sciences, Division of Crop Plant Genetics, Georg-August-University, Göttingen, Germany
† These authors contributed equally to this work.

Corresponding author: Hanna Marie Schilbert; hschilbe@cebitec.uni-bielefeld.de

Theoretical and Applied Genetics

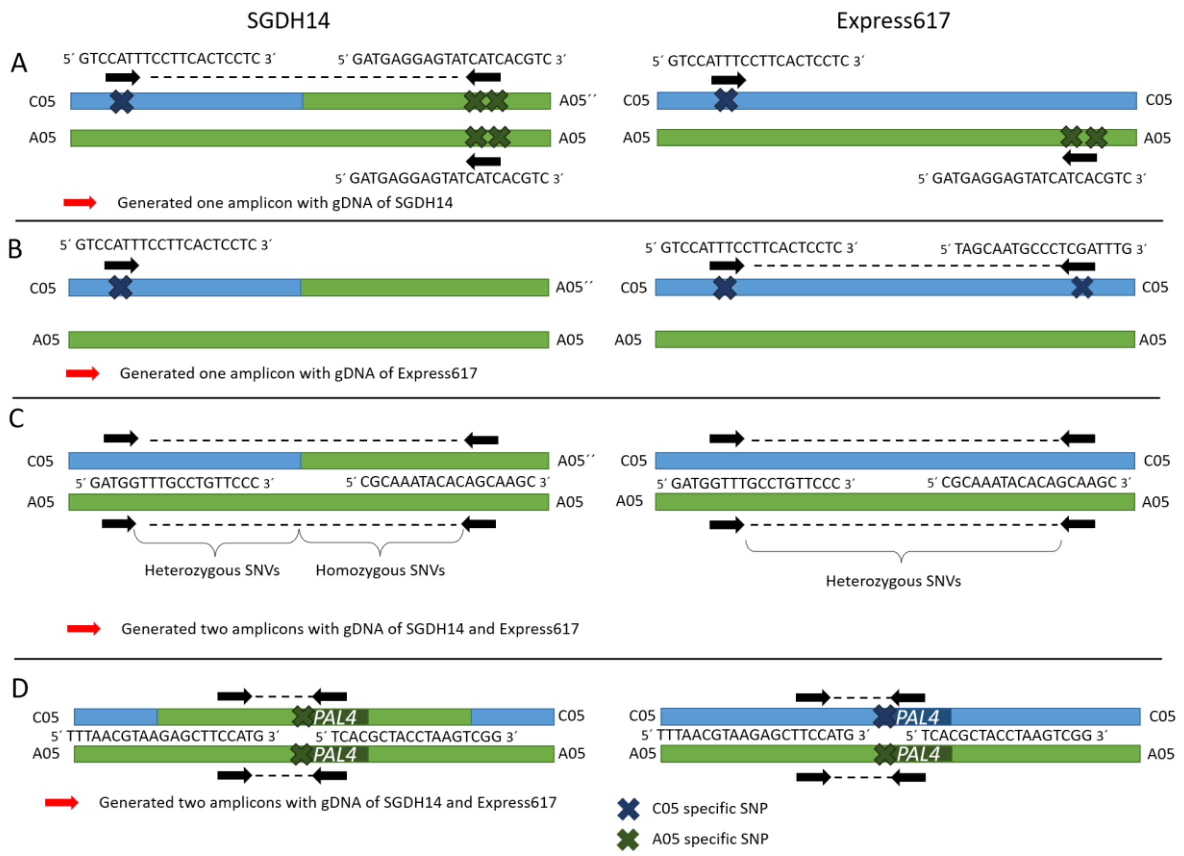

Supplementary Figure 1: Oligonucleotide design strategies for validation of the HNRT border sequences. (A) The subgenome-specific for SGDH14, (B) the subgenomes-specific for Express 617 and (C) not subgenome-specific oligonucleotide design strategies used for the validation of the border sequences are shown, (D) subgenome-specific oligonucleotide design to determine if the *PAL4* gene of the A and/or the C chromosome is present. Oligonucleotides are marked as black arrows. Each strategy was applied for the left and right border, respectively.

Supplementary Table 1: Oligonucleotides used in this study for the three different strategies and for the amplification of *PAL4*. PCR products were sequenced to confirm the correct gene was amplified. The oligonucleotides written in bold and italics indicate the positions which are different between the subgenome-specific primers on the A and C chromosome.

Strategy	Oligonucleotide name	Strand	Sequence (5' to 3')	Annealing Temp. [°C]
A – left border	SGDH14_spec_fw_L	forward	GTC***CA***TTTCCTTCACTCCTC	60
A – left border	SGDH14_spec_rev_L	reverse	GATGAGGAGTATC***AT***CACGTC	
A – right border	SGDH14_spec_fw_R	forward	TCAGA***C***GGC***AG***CGTTTAC	60
A – right border	SGDH14_spec_rev_R	reverse	TTGCCACCACCACC***T***AC	
B – left border	Exp_spec_fw_L	forward	GTC***CA***TTTCCTTCACTCCTC	60
B – left border	Exp_spec_rev_L	reverse	TAGCAATGCCCTC***G***AT***T***TG	
B – right border	Exp_spec_fw_R	forward	TGGTCAGATG***G***C***TC***CGTTTAC	60
B – right border	Exp_spec_rev_R	reverse	TTGCCACCACCACC***T***AC	
C – left border	Not_spec_fw_L	forward	GATGGTTTGCCTGTTCCC	56
C – left border	Not_spec_rev_L	reverse	TCGCTGAATAGTCGCAAG	
C – right border	Not_spec_fw_R	forward	AACCAAATCCGTTGATGC	56
C – right border	Not_spec_rev_R	reverse	TTGCGTGACTGCTCCAAG	
D – PAL4	PAL_nspec_fw_1	forward	TTTAACGTAAGAGCTTCCATG	55
D – PAL4	PAL_nspec_rev_2	reverse	TCACGCTACCTAAGTCGG	

Supplementary Table 2: PCR program for the different subgenomes-specific strategies (A, B) and not subgenome-specific strategy (C), as well as for the amplification of the *PAL4* gene (D).

**Steps**	**Temp. [°C]**	**Time [sec]**	**No. of Cycles**
PCR program for strategy A and C; PAL4			
initial denaturation	95	60	1x
denaturation	95	15	35x
annealing	54, 56, 58, 60	15	
elongation	72	30	
final elongation	72	180	1x
hold	8	infinite	
PCR program for strategy B			
**Steps**	**Temp. [°C]**	**Time [sec]**	**No. of Cycles**
initial denaturation	95	180	1x
denaturation	95	30	15x
annealing	70 _ 0.7°C decrease each cycle	45	
elongation	72	45	
denaturation	95	30	25x
annealing	60 or 61	45	
elongation	72	45	
elongate	72	300	1x
halt reaction	4	300	1x
hold	20	infinite	1x
