# Supplementary material for: Homoeologous non-reciprocal translocation explains a major QTL for seed lignin content in oilseed rape (Brassica napus L.)
Source: Theor Appl Genet. 2023 Jul 13;136(8):172. doi: 10.1007/s00122-023-04407-w (PMC10345078; doi:10.1007/s00122-023-04407-w)
Supplement: Supplementary file 4 — Supplementary file4 (PDF 1244 kb) [file 122_2023_4407_MOESM4_ESM.pdf]

Homoeologous non-reciprocal translocation explains a major QTL for seed lignin content in oilseed rape (*Brassica napus* L.)

Hanna Marie Schilbert[1,2,†], Karin Holzenkamp[3,†], Prisca Viehöver[1], Daniela Holtgräwe[1], Christian Möllers[3]

[1] Genetics and Genomics of Plants, CeBiTec and Faculty of Biology, Bielefeld University, Bielefeld, Germany
[2] Graduate School DILS, Bielefeld Institute for Bioinformatics Infrastructure (BIBI), Faculty of Technology, Bielefeld University, Germany
[3] Department of Crop Sciences, Division of Crop Plant Genetics, Georg-August-University, Göttingen, Germany
† These authors contributed equally to this work.

Corresponding author: Hanna Marie Schilbert; hschilbe@cebitec.uni-bielefeld.de

Theoretical and Applied Genetics

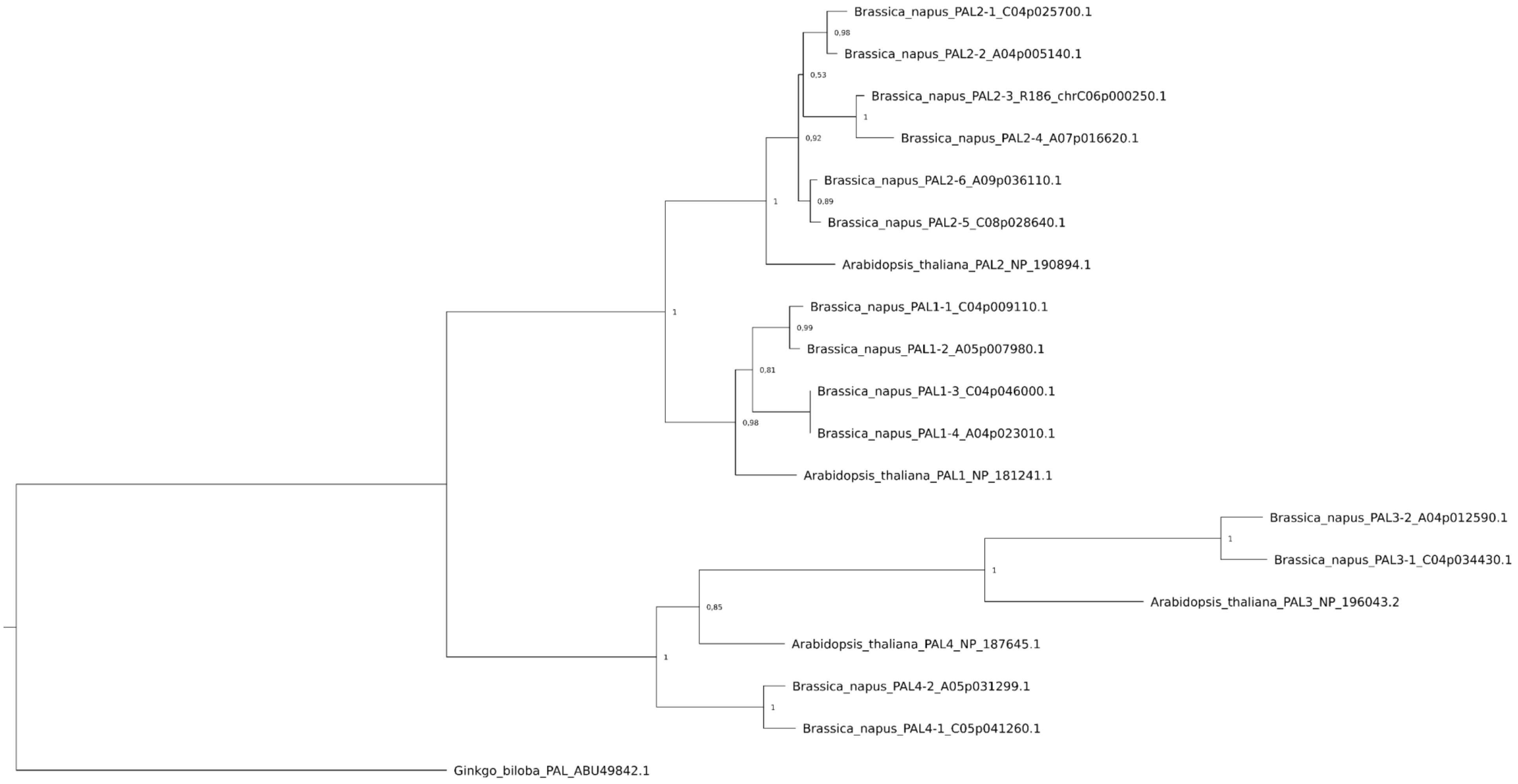

Supplementary Figure 4: Phylogeny of PAL candidates of *B. napus* Express 617 and previously described PAL sequences. The maximum-likelihood tree was constructed with FastTree using 10,000 bootstraps and relative bootstrap-values are shown next to relevant nodes. The phylogenetic tree is based on amino acid sequences. The *Ginkgo biloba* PAL sequence was used to root the tree. The GenBank ID of the respective sequence or the Express 617 gene ID is given for each sequence.

Supplementary Table 6: Amino acid and coding sequences of *B. napus* Express 617 PALs.

>BnaPAL1-4_A04p023010.1
MEVNGLSHGGEVDAMLCGGEIKKNATVVGADPLNWGAAAEQMKGSHLDEVKRMVKEFRRPVVNLGGETLTIGQVAAISTLGNGVKVELSETARAGVKASSDWVMESMGKGTDSYGVTTGFGATSHRRTKN
GVALQKELIRFLNAGIFGSTKETSHTLPHSATRAAMLVRINTLLQGYSGIRFEILEAITSFLNNNITPSLPLRGTITASGDLVPLSYIAGLLTGRPNSKATGPNGEALNAEEAFKMAGVTSGFFDLQPKEGLALVNGT
AVGSGMASMVLFEANVLSVLAEVLSAVFAEVMSGKPEFTDHLTHRLKHHPGQIEAAAIMEHILDGSSYMKLAQKLHEMDPLQKPKQDRYALRTSPQWLGPQIEVIRYATKSIEREINSVNDNPLIDVSRNKAIHG
GNFQGTPIGVSMDNTRLAIAAIGKLMFAQFSELVNDYYNNGLPSNLTASRNPSLDYGFKGAEIAMASYCSELQYLANPVTSHVQSAEQHNQDVNSLGLISSRKTSEAVDILKLMSTTFLVGICQAIDLRHLEENLK
QTVKNTVSQVAKKVLTTGVNGELHPSRFCEKDLLKVVDREQVYTYADDPCSATYPLIQKLRQVIVDHALVNGESEKNAMTSIFHKIGAFEEELKAVLPKEVEAARAAYDNGTAAIPNRIKECRSYPLYRFVREEL
GTELLTGEKATSPGEEFDKVFTAICEGKIIDPLMECLDEWNGAPIPIC*

>BnaPAL1-4_A04p023010.1_BnaEXP
ATGGAGGTTAACGGATTATCACACGGAGGAGAAGTCGACGCTATGTTATGCGGCGGAGAGATCAAGAAGAATGCCACGGTGGTTGGGGCTGATCCTCTCAACTGGGGAGCTGCAGCGGAGCAAATG
AAAGGGAGCCATTTGGATGAGGTGAAGAGAATGGTGAAGGAGTTTAGGAGGCCAGTGGTGAATCTCGGAGGCGAGACTCTCACGATCGGACAAGTGGCAGCAATCTCGACCCTTGGAAACGGTGTG
AAGGTGGAGTTGTCGGAGACGGCGAGAGCCGGTGTGAAAGCGAGTAGTGATTGGGTGATGGAGAGTATGGGCAAAGGAACCGATAGTTATGGTGTTACTACTGGTTTTGGTGCTACTTCTCACCGGA
GAACCAAGAACGGTGTCGCTCTTCAGAAAGAGCTTATAAGATTTCTGAACGCCGGAATATTCGGCAGCACGAAGGAAACATCCCACACATTACCACACTCCGCCACAAGAGCCGCCATGCTCGTACGC
ATCAACACTCTCCTCCAAGGCTACTCAGGCATACGTTTCGAGATCCTCGAGGCCATAACAAGTTTCCTCAACAACAACATCACTCCTTCACTCCCTCTCCGCGGCACCATCACCGCCTCCGGAGACCT
CGTCCCCCTCTCCTACATCGCCGGACTCTTAACCGGACGTCCCAACTCCAAAGCCACCGGTCCCAACGGCGAAGCCTTGAACGCAGAGGAAGCCTTCAAAATGGCAGGAGTCACCTCCGGCTTCTTC
GACCTCCAGCCTAAAGAAGGCCTCGCCCTAGTCAACGGCACGGCCGTTGGCTCCGGCATGGCGTCGATGGTTCTCTTCGAAGCCAACGTCCTCTCGGTCCTGGCCGAGGTTTTATCAGCCGTCTTC
GCCGAGGTGATGAGCGGGAAGCCGGAGTTCACCGATCATTTGACTCACCGGCTGAAGCACCACCCCGGGCAGATAGAAGCCGCGGCTATAATGGAACACATTCTCGACGGGAGCTCGTACATGAAG
CTAGCTCAGAAGCTTCACGAGATGGATCCGTTACAAAAACCCAAACAAGACCGTTACGCTCTACGTACCTCTCCCCAATGGCTAGGTCCTCAGATAGAAGTGATCCGTTACGCGACCAAGTCCATCGA
GCGTGAGATAAACTCCGTCAACGACAACCCTTTGATCGATGTCTCGAGAAACAAAGCCATTCACGGTGGTAACTTCCAGGGAACACCCATCGGTGTGTCTATGGACAACACACGTTTAGCTATCGCCGG
CCATAGGGAAGCTCATGTTCGCTCAGTTCTCCGAGCTGGTCAACGATTATTACAACAACGGTCTTCCTTCGAACCTAACCGCTTCGAGGAACCCTAGTTTGGACTACGGTTTCAAAGGAGCGGAGATC
GCGATGGCTTCGTACTGTTCAGAGCTTCAGTACTTGGCCAACCCCGTGACTAGCCATGTTCAATCAGCAGAGCAACACAACCAAGACGTGAACTCTTTAGGACTAATCTCATCTCGCAAGACATCAGAA
GCTGTAGACATACTCAAACTCATGTCGACAACGTTCCTTGTCGGAATCTGCCAAGCCATCGATCTAAGACACTTGGAGGAGAATCTAAAACAAACGGTCAAGAACACAGTCTCTCAAGTCGCCAAGAAA
GTTCTCACCACTGGAGTCAACGGTGAGCTTCACCCATCACGCTTCTGCGAGAAAGACTTACTCAAAGTCGTCGACCGTGAGCAAGTCTACACGTACGCGGACGACCCATGCAGCGCCACGTACCCGT
TGATCCAGAAGCTGAGGCAAGTCATCGTTGACCATGCTTTGGTCAACGGTGAGAGCGAGAAGAATGCAATGACTTCGATCTTCCATAAGATTGGAGCCTTCGAGGAGGAGCTCAAGGCGGTGCTACC
TAAAGAAGTGGAGGCAGCTAGAGCGGCGTATGATAACGGAACAGCTGCGATACCGAACAGGATCAAGGAGTGTAGGTCGTATCCTTTGTATAGGTTTGTGAGGGAGGAGCTTGGAACAGAGCTTTTG
ACCGGAGAGAAAGCGACGTCGCCCGGAGAAGAGTTTGATAAGGTTTTCACGGCGATTTGTGAAGGGAAGATTATTGATCCGTTGATGGAGTGTCTCGATGAGTGGAACGGAGCTCCCATTCCTATAT
GTTAA

>BnaPAL1-3_C04p046000.1
MEVNGLSHGGEVDAMLCGGEIKKNATVVGADPLNWGAAAEQMKGSHLDEVKRMVKEFRRPVVNLGGETLTIGQVAAISTLGNGVKVELSETARAGVKASSDWVMESMGKGTDSYGVTTGFGATSHRRTKN
GVALQKELIRFLNAGIFGSTKETSHTLPHSATRAAMLVRINTLLQGYSGIRFEILEAITSFLNNNITPSLPLRGTITASGDLVPLSYIAGLLTGRPNSKATGPNGEALNAEEAFKMAGVTSGFFDLQPKEGLALVNGT
AVGSGMASMVLFEANVLSVLAEVLSAVFAEVMSGKPEFTDHLTHRLKHHPGQIEAAAIMEHILDGSSYMKLAQKLHEMDPLQKPKQDRYALRTSPQWLGPQIEVIRYATKSIEREINSVNDNPLIDVSRNKAIHG
GNFQGTPIGVSMDNTRLAIAAIGKLMFAQFSELVNDYYNNGLPSNLTASRNPSLDYGFKGAEIAMASYCSELQYLANPVTSHVQSAEQHNQDVNSLGLISSRKTSEAVDILKLMSTTFLVGICQAIDLRHLEENLK
QTVKNTVSQVAKKVLTTGVNGELHPSRFCEKDLLKVVDREQVYTYADDPCSATYPLIQKLRQVIVDHALVNGESEKNAMTSIFHKIGAFEEELKAVLPKEVEAARAAYDNGTAAIPNRIKECRSYPLYRFVREEL
GTELLTGEKATSPGEEFDKVFTAICEGKIIDPLMECLDEWNGAPIPIC*

>BnaPAL1-3_C04p046000.1_BnaEXP
ATGGAGGTTAACGGATTATCACACGGAGGAGAAGTCGACGCTATGTTATGCGGCGGAGAGATCAAGAAGAATGCCACGGTGGTTGGGGCTGATCCTCTCAACTGGGGAGCTGCAGCGGAGCAAATG
AAAGGGAGCCATTTGGATGAGGTGAAGAGAATGGTGAAGGAGTTTAGGAGGCCAGTGGTGAATCTCGGAGGCGAGACTCTCACGATCGGACAAGTGGCAGCAATCTCGACCCTTGGAAACGGTGTG
AAGGTGGAGTTGTCGGAGACGGCGAGAGCCGGTGTGAAAGCGAGTAGTGATTGGGTGATGGAGAGTATGGGCAAAGGAACCGATAGTTATGGTGTTACTACTGGTTTTGGTGCTACTTCTCACCGGA
GAACCAAGAACGGTGTCGCTCTTCAGAAAGAGCTTATAAGATTTCTGAACGCCGGAATATTCGGCAGCACGAAGGAAACATCCCACACATTACCACACTCCGCCACAAGAGCCGCCATGCTCGTACGT
ATCAACACTCTCCTCCAAGGATACTCCGGCATACGTTTCGAGATCCTCGAGGCCATAACAAGTTTCCTCAACAACAACATCACTCCTTCCCTCCCTCTCCGCGGCACCATCACCGCCTCCGGAGACCT
CGTCCCCCTCTCCTACATCGCCGGACTCTTAACCGGACGTCCCAACTCCAAAGCTACCGGTCCCAACGGCGAAGCCCTAAACGCAGAGGAAGCCTTCAAAATGGCTGGAGTCACCTCAGGCTTCTTT
GACCTCCAGCCTAAGGAAGGCCTCGCCCTAGTCAACGGCACGGCCGTTGGCTCCGGCATGGCGTCGATGGTTCTCTTCGAAGCCAACGTCCTCTCGGTCCTGGCCGAGGTTTTATCAGCCGTCTTC
GCCGAGGTGATGAGCGGGAAGCCGGAGTTCACCGATCATTTGACTCACCGGCTGAAGCACCACCCCGGGCAGATAGAAGCCGCGGCTATAATGGAACACATTCTCGACGGGAGCTCGTACATGAAG
CTAGCTCAGAAGCTTCACGAGATGGATCCGTTACAAAAACCCAAACAAGACCGTTACGCTCTACGTACCTCTCCCCAATGGCTAGGTCCTCAGATAGAAGTGATCCGTTACGCGACCAAGTCCATCGA
GCGTGAGATAAACTCCGTCAACGACAACCCTTTGATCGATGTCTCGAGAAACAAAGCCATTCACGGTGGTAACTTCCAGGGAACACCCATCGGTGTGTCTATGGACAACACACGTTTAGCTATCGCGG
CCATAGGGAAGCTCATGTTCGCTCAGTTCTCCGAGCTGGTCAACGATTATTACAACAACGGTCTTCCTTCGAACCTAACCGCTTCGAGGAACCCTAGTTTGGACTACGGTTTCAAAGGAGCGGAGATC

GCGATGGCTTCGTACTGTTCAGAGCTTCAGTACTTGGCCAACCCAGTGACTAGCCATGTTCAATCAGCTGAGCAACACAACCAAGACGTGAACTCTTTAGGACTAATCTCGTCTCGCAAGACATCAGAA
GCTGTAGACATACTCAAACTCATGTCTACAACGTTCCTTGTCGGAATCTGCCAAGCCATCGATCTAAGACACTTGGAGGAGAATCTAAAACAAACGGTCAAGAACACAGTCTCTCAAGTCGCCAAGAAA
GTTCTCACCACTGGAGTCAACGGTGAGCTTCACCCATCACGCTTCTGCGAGAAAGACTTACTCAAAGTCGTCGACCGTGAGCAAGTCTACACGTACGCGGACGACCCATGCAGCGCCACGTACCCGT
TGATCCAGAAGCTGAGGCAAGTCATCGTTGACCATGCTTTGGTCAACGGTGAGAGCGAGAAGAATGCAATGACTTCGATCTTCCATAAGATTGGAGCCTTCGAGGAGGAGCTCAAGGCGGTGCTTCC
GAAAGAAGTGGAGGCAGCTAGAGCGGCGTATGATAACGGAACAGCTGCGATACCGAACAGGATCAAGGAGTGTAGGTCGTATCCTTTGTATAGGTTTGTGAGGGAGGAGCTTGGAACAGAGCTTTTG
ACCGGAGAGAAAGCGACGTCGCCCGGAGAAGAGTTTGATAAGGTTTTCACGGCGATCTGTGAAGGGAAGATTATTGATCCGTTGATGGAGTGTCTCGATGAGTGGAACGGAGCTCCCATTCCTATAT
GTTAA

>BnaPAL1-2_A05p007980.1
MEINGSSHKVDAMLCGAEIKNVTVAAADPLNWGAAAEQMKGSHLDEVKRMVMEFRKPVVNLGGETLTIGQVAAISTVGNGVKVELSETARAGVKASSDWVMESMGKGTDSYGVTTGFGATSHRRTKNGVAL
QKELIRFLNAGIFGSTKETCHTLPHSATRAAMLVRINTLLQGYSGIRFEILEAITSFLNTNITPSLPLRGTITASGDLVPLSYIAGLLTGRPNSKATGPNGEALNAEEAFKKAGIPSGFFDLQPKEGLALVNGTAVGSG
MASMVLFETNVLSVLAEVLSAVFAEVMSGKPEFTDHLTHRLKHHPGQIEAAAIMEHILDGSSYMKLAKKLHELDPLQKPKQDRYALRTSPQWLGPQIEVIRYATKSIEREINSVNDNPLIDVSRNKAIHGGNFQGT
PIGVSMDNTRLAIASIGKLMFAQFSELVNDFYNNGLPSNLTASRNPSLDYGFKGAEIAMASYCSELQYLANPVTTHVQSAEQHNQDVNSLGLISSRKTAEAVDILKLMSTTFLVAICQAVDLRHLEENLKQTVKNT
VSQVAKKVLTTGVNGELHPSRFCEKDLLKVVDREQVYTYADDPCSATYPLIQKLRQVIVDHALVNGESEKNAMTSIFHKIGAFEEELKAVLPDEVEAARVAYDNGTSAIPNRIKECRSYPLYRFVREELGTELLTG
EKVTSPGEEFDKVFTAICEGKIIDPLMECLSEWNGAPIPIC*

>BnaPAL1-2_A05p007980.1_BnaEXP
ATGGAGATTAACGGATCATCACACAAAGTCGACGCTATGTTATGCGGCGCAGAGATCAAGAATGTCACGGTGGCTGCGGCGGATCCTCTCAACTGGGGGGCTGCGGCGGAGCAAATGAAAGGGAGC
CATTTGGATGAAGTGAAGAGAATGGTTATGGAGTTTAGGAAGCCTGTGGTGAATCTCGGAGGAGAGACTCTGACGATCGGACAAGTAGCAGCGATCTCGACCGTTGGAAATGGTGTGAAGGTGGAGC
TGTCGGAGACGGCAAGAGCCGGTGTGAAGGCGAGCAGTGATTGGGTGATGGAGAGTATGGGCAAAGGCACTGATAGTTATGGTGTTACTACTGGTTTTGGTGCTACTTCTCATCGTAGAACCAAAAA
TGGCGTCGCACTTCAGAAGGAGCTTATAAGATTCCTGAACGCCGGAATATTCGGCAGCACGAAAGAAACATGCCACACACTACCACACTCCGCCACGAGAGCCGCCATGCTCGTACGCATCAACACT
CTCCTCCAAGGATACTCCGGCATCCGCTTCGAGATCCTCGAGGCCATAACCAGCTTCCTCAACACCAACATCACTCCTTCCCTCCCTCTCCGCGGCACCATCACCGCCTCCGGAGACCTCGTCCCCC
TCTCCTACATCGCCGGCCTCCTCACCGGCCGTCCCAACTCCAAAGCCACCGGTCCCAACGGCGAAGCCTTGAACGCAGAGGAAGCCTTCAAGAAGGCTGGAATCCCCTCCGGATTCTTTGACTTGCA
GCCCAAGGAAGGTCTCGCCCTAGTCAACGGCACAGCCGTTGGCTCCGGCATGGCCTCGATGGTGCTCTTCGAGACGAACGTCCTCTCGGTCCTGGCCGAGGTTCTCTCAGCTGTCTTCGCCGAGGT
GATGAGCGGAAAGCCGGAGTTCACCGACCATTTAACCCACAGGCTTAAACACCACCCCGGCCAGATCGAAGCCGCGGCGATAATGGAGCACATCCTTGACGGGAGCTCGTACATGAAGCTAGCTAA
GAAGCTACACGAGTTGGATCCGTTACAGAAACCTAAACAAGACCGTTACGCGCTCCGCACGTCACCTCAATGGCTAGGTCCTCAGATAGAAGTGATCCGTTACGCAACAAAGTCCATCGAACGTGAGA
TCAACTCCGTCAACGACAACCCCTTGATCGACGTTTCGAGAAACAAGGCCATTCACGGAGGTAACTTCCAGGGAACACCGATAGGTGTCTCCATGGACAACACGCGTCTCGCTATAGCATCGATAGG
GAAGCTCATGTTCGCTCAGTTCTCTGAGCTCGTCAACGACTTCTACAACAACGGTCTTCCTTCTAACCTAACCGCCTCGAGGAACCCTAGTTTGGACTACGGTTTCAAAGGAGCGGAGATCGCTATGG
CATCCTACTGTTCCGAGCTTCAGTACTTAGCCAATCCAGTTACAACCCATGTTCAATCCGCGGAGCAACACAACCAAGACGTGAACTCTTTAGGACTAATCTCGTCTCGCAAGACGGCAGAAGCTGTTG
ACATTCTCAAACTCATGTCGACGACGTTCCTTGTCGCAATCTGCCAAGCTGTAGATCTAAGACACTTGGAGGAGAATCTAAAACAAACCGTGAAGAACACGGTCTCTCAAGTGGCTAAGAAAGTTCTAA
CCACTGGAGTCAACGGTGAGCTTCACCCATCTCGCTTCTGCGAGAAAGATTTACTCAAAGTCGTTGACCGTGAACAAGTCTACACGTACGCGGACGATCCATGCAGCGCTACGTACCCGTTGATCCAG
AAGCTAAGGCAAGTTATCGTTGACCATGCTTTGGTCAACGGTGAGAGTGAGAAGAACGCTATGACTTCGATCTTCCACAAGATTGGTGCTTTCGAGGAGGAGCTCAAGGCGGTGCTTCCTGATGAAGT
GGAGGCAGCTAGAGTGGCGTACGATAACGGGACATCTGCTATACCGAACCGGATCAAGGAGTGTAGGTCGTATCCTTTGTATAGGTTCGTGAGGGAGGAGCTTGGAACTGAGCTTTTGACCGGAGAG
AAAGTCACGTCGCCGGGAGAAGAGTTTGATAAGGTTTTCACGGCGATATGTGAAGGGAAGATCATTGATCCGTTGATGGAGTGTCTCAGTGAGTGGAACGGAGCTCCCATTCCAATCTGCTAA

>BnaPAL1-1_C04p009110.1
MEINGSSYKVDAMLCGGETKNVTVAAADPLNWGAAADQMKGSHLDEVKRMVMEFRKPVVNLGGETLTIGQVAAISTVGNGVKVELSETARAGVKASSDWVMESMGKGTDSYGVTTGFGATSHRRTKNGVA
LQKELIRFLNAGIFGSTKETCHTLPHSATRAAMLVRINTLLQGYSGIRFEILEAITSFLNTNITPSLPLRGTITASGDLVPLSYIAGLLTGRPNSKATGPNGEALNAEEAFKKAGIPSGFFDLQPKEGLALVNGTAVGS
GMASMVLFETNVLSVLAEVLSAVFAEVMSGKPEFTDHLTHRLKHHPGQIEAAAIMEHILDGSSYMKLAQKLHEMDPLQKPKQDRYALRTSPQWLGPQIEVIRYATKSIEREINSVNDNPLIDVSRNKAIHGGNFQ
GTPIGVSMDNTRLAIASIGKLMFAQFSELVNDFYNNGLPSNLTASRNPSLDYGFKGAEIAMASYCSELQYLANPVTTHVQSAEQHNQDVNSLGLISSRKTAEAVDILKLMSTTFLVAICQAVDLRHLEENLKQTVK
NTVSQVAKKVLTTGDNGELHPSRFCEKDLLKVVDREQVYTYADDPCSATYPLIQKLRQVIVDHALVNGESEKNAMTSIFHKIGAFEEELKAVLPDEVEAARVAYDNGTSAIPNRIKECRSYPLYRFVREELGTELL
TGEKVTSPGEEFDKVFTAICEGKIIDPLMECLSEWNGAPIPIC*

>BnaPAL1-1_C04p009110.1_BnaEXP
ATGGAGATTAACGGATCATCATACAAAGTCGACGCTATGTTATGCGGCGGAGAGACCAAGAATGTCACGGTGGCTGCGGCGGATCCTCTGAACTGGGGAGCTGCGGCGGATCAAATGAAAGGGAGC
CATTTGGATGAAGTGAAGAGAATGGTTATGGAGTTTAGGAAGCCTGTGGTGAATCTCGGAGGAGAGACTCTGACGATCGGACAAGTAGCAGCGATCTCGACCGTTGGAAATGGTGTGAAGGTGGAGC
TGTCGGAGACGGCAAGAGCCGGTGTGAAGGCGAGCAGTGATTGGGTGATGGAGAGTATGGGCAAAGGCACTGATAGTTATGGTGTTACTACTGGTTTTGGTGCTACTTCTCATCGTAGAACCAAAAA
TGGCGTCGCACTTCAGAAGGAGCTTATAAGATTCCTGAACGCCGGAATATTCGGCAGCACGAAAGAAACATGCCACACACTGCCACACTCCGCCACAAGAGCTGCCATGCTCGTACGCATAAACACT
CTCCTCCAAGGATACTCCGGCATCCGCTTCGAGATCCTCGAGGCCATAACCAGCTTCCTCAACACCAACATCACTCCTTCCCTCCCTCTCCGCGGCACCATCACCGCCTCCGGAGACCTCGTCCCCC
TCTCCTACATCGCCGGCCTCCTCACCGGCCGTCCCAACTCCAAAGCCACCGGTCCCAACGGCGAAGCCTTGAACGCAGAGGAAGCCTTCAAGAAGGCCGGAATCCCCTCTGGATTCTTTGACTTGCA

GCCCAAGGAAGGTCTCGCCCTAGTCAACGGCACAGCCGTTGGCTCCGGCATGGCCTCGATGGTTCTCTTCGAGACAAACGTCCTGTCGGTTCTGGCCGAGGTTCTCTCAGCTGTCTTCGCCGAGGT<br>GATGAGCGGCAAGCCGGAGTTCACCGACCATTTAACCCACAGGCTGAAGCACCACCCCGGCCAGATCGAAGCCGCGGCGATAATGGAGCACATCCTCGACGGGAGCTCGTACATGAAGCTAGCTCA<br>GAAGCTACACGAGATGGATCCGTTACAGAAACCTAAACAAGACCGTTACGCGCTCCGCACATCACCTCAATGGCTAGGTCCTCAGATAGAAGTGATCCGTTACGCCACCAAGTCCATCGAGCGTGAG<br>ATAAACTCCGTCAACGACAACCCTTTGATCGACGTTTCAAGAAACAAAGCGATTCACGGAGGTAACTTCCAGGGGACACCCATCGGTGTCTCCATGGACAACACGCGTCTCGCTATAGCATCGATAGG<br>GAAGCTCATGTTCGCTCAGTTCTCCGAGCTTGTTAACGACTTCTACAACAACGGTCTTCCTTCTAACCTAACCGCTTCGAGGAACCCTAGTTTGGACTACGGTTTCAAAGGAGCGGAGATCGCTATGGC<br>TTCCTACTGTTCAGAGCTTCAGTACTTGGCCAATCCAGTTACAACCCATGTTCAGTCAGCAGAGCAACACAACCAAGACGTGAACTCTTTAGGACTAATCTCATCACGCAAGACGGCAGAAGCTGTCGA<br>CATACTCAAACTCATGTCGACAACGTTCCTCGTCGCAATCTGCCAAGCTGTGGATCTAAGACACTTGGAGGAGAATCTAAAACAAACCGTGAAGAACACTGTCTCTCAAGTGGCCAAGAAAGTTCTAAC<br>CACTGGAGACAACGGTGAGCTTCACCCATCTCGCTTCTGCGAGAAAGATTTACTCAAAGTCGTTGACCGTGAACAAGTCTACACGTACGCGGACGATCCATGCAGCGCCACGTACCCGTTGATCCAG<br>AAGCTAAGGCAAGTTATCGTTGACCATGCTTTGGTCAACGGTGAGAGCGAGAAGAACGCTATGACTTCGATCTTCCACAAGATTGGTGCTTTCGAGGAGGAGCTCAAGGCGGTGCTTCCTGACGAAGT<br>GGAGGCAGCTCGAGTGGCGTATGATAACGGGACATCTGCTATACCGAACCGGATCAAGGAGTGTAGGTCGTATCCTTTGTATAGGTTCGTGAGGGAGGAGCTTGGAACTGAGCTTTTGACCGGAGAG<br>AAGGTGACGTCGCCGGGAGAAGAGTTTGATAAGGTTTTCACGGCGATATGTGAAGGGAAGATCATTGATCCGTTGATGGAGTGTCTCAGTGAGTGGAACGGAGCTCCCATTCCAATATGCTAA
>BnaPAL2-2_A04p005140.1<br>MDQINGSVHQNGKTEAMLLCGGLEKTKVTVAADPLNWGAAAEQMKGSHLDEVKRMVEDYRKPVVNLGGETLTIGQVAAISNVGGGVKVELAEASRAGVKASSDWVMESMGKGTDSYGVTTGFGATSHRRT<br>KNGTALQTELIRFLNAGIFGNTKETCHTLPESATRAAMLVRVNTLLQGYSGIRFEILEAITSLLNHNISPSLPLRGTITASGDLVPLSYIAGLLTGRPNSKATGPNGESLTAEEAFKKAGITSGFFDLQPKEGLALVNG<br>TAVGSGMASMVLFEANVQSVLAEVLSAIFAEVMSGKPEFTDHLTHRLKHHPGQIEAAAIMEHILDGSSYMKLAAKLHEMDPLQKPKQDRYALRTSPQWLGPQIEVIRHATKSIEREINSVNDNPLIDVSRNKAIHG<br>GNFQGTPIGVSMDNTRLAVAAIGKLMFAQFSELVNDFYNNGLPSNLTASNNPSLDYGFKGAEIAMASYCSELQYLANPVTTHVQSAEQHNQDVNSLGLISSRKTSEAVDILKLMSTTFLVAICQAVDLRHLEENL<br>RQTVKNTVSQVAKKVLTTGVNGELHPSRFCEKDLLKVVDREQVFTYVDDPCSATYPLMQKLRQVIVDHALSNGETEKNAVTSIFQKIGAFEEELKMVLPKEVDATREAYGNGTAAIPNRIKECRSYPLYKFVREE<br>LGTKLLTGEKVVSPGEEFDKVFTAMCEGKIIDPLMDCLKEWNGAPIPIC*
>BnaPAL2-2_A04p005140.1_BnaEXP<br>ATGGATCAGATCAACGGATCAGTTCACCAAAACGGCAAAACCGAAGCCATGTTGTTGTGCGGCGGACTAGAGAAGACTAAAGTGACGGTGGCGGCGGATCCGTTGAATTGGGGTGCTGCGGCGGAG<br>CAGATGAAAGGGAGTCATTTGGATGAGGTGAAGAGGATGGTTGAGGATTACCGTAAACCGGTGGTGAATCTCGGCGGAGAAACACTGACGATCGGACAAGTCGCTGCGATCTCGAACGTAGGCGGT<br>GGCGTTAAGGTTGAGCTAGCGGAGGCTTCAAGAGCCGGCGTGAAAGCTAGCAGCGATTGGGTCATGGAGAGTATGGGTAAAGGTACTGACAGTTACGGTGTAACCACCGGGTTTGGAGCTACCTCT<br>CACCGGAGAACAAAAAACGGCACCGCTTTGCAAACCGAACTCATCAGGTTTTTGAACGCCGGAATATTCGGGAACACGAAGGAGACATGCCACACGCTACCGGAATCCGCCACGAGAGCCGCCATG<br>CTCGTCCGAGTCAACACACTCCTCCAAGGATACTCCGGGATCCGATTCGAAATCCTCGAAGCGATCACCAGCCTCCTCAACCACAACATCTCTCCGTCTCTCCCCCTCCGTGGAACCATAACCGCCTC<br>CGGCGATCTCGTCCCCCTCTCCTACATCGCCGGTCTCCTCACCGGCCGTCCAAACTCCAAAGCCACCGGTCCCAACGGCGAATCCCTAACCGCCGAGGAAGCCTTCAAGAAAGCGGGAATCACTTC<br>CGGATTCTTCGATCTACAGCCTAAGGAAGGCTTAGCGCTCGTCAACGGCACGGCGGTTGGATCCGGCATGGCGTCGATGGTTCTCTTCGAAGCGAATGTTCAATCGGTCCTCGCCGAGGTTTTATCC<br>GCGATCTTCGCGGAGGTGATGAGCGGGAAGCCGGAGTTCACCGATCATTTGACTCACCGACTCAAACACCACCCCGGACAAATCGAGGCGGCGGCGATCATGGAGCACATCCTCGACGGAAGCTCG<br>TACATGAAGCTAGCGGCAAAGCTCCACGAGATGGATCCGTTACAAAAACCGAAACAAGACCGTTACGCGCTCCGCACGTCTCCTCAGTGGCTAGGCCCTCAGATCGAAGTGATCCGTCACGCCACGA<br>AGTCAATCGAGCGTGAGATCAACTCCGTTAATGATAATCCGTTAATCGACGTTTCGAGGAACAAAGCGATTCACGGTGGTAACTTCCAGGGGACTCCGATAGGAGTCTCTATGGACAACACACGTTTA<br>GCGGTTGCAGCTATTGGGAAGCTCATGTTCGCTCAGTTCTCGGAGCTTGTTAACGACTTCTACAACAACGGTCTTCCTTCGAATCTAACGGCTTCCAACAACCCAAGCTTGGATTACGGATTCAAAGGA<br>GCGGAGATCGCTATGGCTTCTTACTGCTCTGAGCTTCAGTACCTTGCGAATCCGGTTACAACCCATGTTCAATCAGCTGAGCAGCATAACCAAGATGTTAACTCTTTAGGACTCATCTCGTCTCGTAAG<br>ACGTCAGAAGCTGTTGACATTCTCAAGCTCATGTCTACAACGTTCCTCGTAGCGATTTGTCAAGCTGTTGATTTGAGACATCTCGAGGAGAATCTGAGACAGACCGTGAAGAACACGGTTTCTCAAGTG<br>GCGAAGAAAGTGTTGACTACTGGAGTCAACGGGGAGTTGCATCCATCGCGGTTCTGTGAGAAAGACTTGCTTAAGGTCGTTGATCGTGAACAAGTGTTTACATACGTTGACGATCCTTGTAGCGCTAC<br>GTACCCATTGATGCAGAAGCTAAGACAAGTTATCGTTGATCACGCGTTATCCAATGGTGAGACTGAGAAGAACGCAGTGACTTCGATCTTTCAAAAGATTGGAGCTTTTGAGGAGGAGCTTAAGATGGT<br>GCTTCCTAAGGAAGTGGATGCGACTAGAGAGGCTTACGGTAATGGAACGGCGGCGATTCCGAACAGGATTAAGGAATGTCGGTCTTATCCATTATATAAGTTTGTGAGGGAAGAGCTCGGGACGAAG<br>TTGCTGACCGGAGAAAAGGTTGTCTCTCCGGGAGAGGAGTTTGATAAGGTGTTCACTGCAATGTGTGAAGGTAAGATTATTGATCCATTGATGGATTGTCTCAAGGAATGGAACGGAGCTCCCATTCC<br>CATATGTTAA
>BnaPAL2-6_A09p036110.1<br>MDQINSSVHQNGKIEAMLCGGVEKTKVAVAADPLNWGAAAEQMKGSHLDEVKRMVEEYRRPVVNLGGETLTIGQVAAISTVGGGVKVELAEASRAGVKASSDWVMESMGKGTDSYGVTTGFGATSHRRTK<br>NGTALQTELIRFLNAGIFGNTKETCHTLPESATRAAMLVRVNTLLQGYSGIRFEILEAITSFLNHNISPSLPLRGTITASGDLVPLSYIAGLLTGRPNSKATGPNGESLTAEEAFKQAGIASGFFDLQPKEGLALVNG<br>TAVGSGMASMVLFEANVQSVLAEVLSAIFAEVMSGKPEFTDHLTHRLKHHPGQIEAAAIMEHILDGSSYMKLAQKLHEMDPLQKPKQDRYALRTSPQWLGPQIEVIRHATKSIEREINSVNDNPLIDVSRNKAIHG<br>GNFQGTPIGVSMDNTRLAIASIGKLMFAQFSELVNDFYNNGLPSNLTASNNPSLDYGFKGAEIAMASYCSELQYLANPVTSHVQSAEQHNQDVNSLGLISSRKTSEAVDILKLMSTTFLVAICQAVDLRHLEENL<br>RQTVKNTVSQVAKKVLTTGVNGELHPSRFCEKDLLKVVDREQVFTYVDDPCLATYPLMQKLRQVIVDHALSNGETEKNAVTSIFQKIGAFEEELKMVLPKEVDAAREAYGNGTAAIPNRIKECRSYPLYKFVREE<br>LGTKLLTGEKVVSPGEEFDKVFTAMCEGKIIDPLMECLKEWNGAPIPIC*
>BnaPAL2-6_A09p036110.1_BnaEXP<br>ATGGATCAGATTAACAGTTCAGTTCACCAGAACGGCAAGATCGAAGCCATGTTGTGCGGCGGAGTAGAGAAGACGAAGGTGGCTGTGGCGGCGGATCCGTTGAACTGGGGTGCAGCAGCGGAGCA

GATGAAAGGGAGTCACTTGGATGAGGTGAAGAGGATGGTTGAGGAGTATCGTAGACCGGTGGTGAATCTCGGAGGAGAGACGCTAACAATCGGACAAGTCGCGGCGATCTCCACCGTAGGCGGAG
GTGTTAAGGTTGAGCTAGCGGAGGCTTCGAGAGCCGGCGTGAAAGCTAGCAGTGATTGGGTTATGGAGAGTATGGGCAAAGGTACTGACAGTTACGGTGTCACCACCGGGTTTGGGGCGACCTCTC
ACCGGAGAACCAAAAACGGCACCGCATTGCAAACCGAACTCATCAGATTTTTGAACGCCGGAATATTCGGTAACACGAAGGAGACATGCCACACGCTACCGGAATCCGCCACGAGAGCCGCCATGCT
CGTCCGAGTCAACACTCTCCTCCAAGGATACTCCGGGATCCGATTCGAAATCCTCGAAGCGATCACCAGCTTCCTCAACCACAACATCTCTCCGTCTCTCCCCCTCCGCGGAACCATAACCGCCTCCG
GCGATCTCGTCCCCCTCTCCTACATCGCCGGCCTCCTCACCGGCCGTCCGAACTCCAAAGCCACAGGTCCCAACGGCGAATCCCTAACCGCCGAAGAGGCCTTCAAACAAGCCGGAATCGCTTCCG
GATTCTTCGATCTACAGCCTAAAGAAGGCTTAGCGCTCGTTAACGGCACGGCGGTTGGATCCGGCATGGCGTCGATGGTTCTATTCGAAGCGAACGTTCAATCGGTGTTAGCGGAGGTCTTATCAGC
GATCTTCGCGGAGGTTATGAGCGGGAAGCCTGAGTTCACCGATCATCTGACTCACAGGCTGAAACACCACCCCGGACAAATCGAAGCGGCGGCGATCATGGAGCACATCCTCGACGGAAGCTCGTA
CATGAAGCTAGCGCAAAAGCTTCACGAGATGGATCCGTTACAAAAACCGAAGCAAGACCGTTACGCTCTCCGTACCTCTCCTCAGTGGCTCGGCCCTCAGATCGAAGTGATCCGTCACGCCACGAAG
TCGATCGAGCGTGAGATCAACTCCGTTAACGATAATCCGTTGATCGATGTTTCTAGAAACAAAGCGATTCACGGTGGTAACTTCCAGGGGACTCCGATCGGAGTCTCCATGGATAACACGCGTTTAGC
GATCGCTTCGATAGGGAAGCTCATGTTCGCTCAATTCTCGGAGCTTGTTAACGATTTCTATAACAATGGCCTTCCTTCGAATCTAACGGCTTCGAACAATCCAAGCTTGGATTACGGATTCAAAGGAGC
GGAGATCGCTATGGCTTCGTATTGCTCTGAGCTTCAGTACTTGGCGAATCCAGTTACAAGCCATGTTCAATCAGCTGAGCAACATAACCAAGATGTTAACTCTTTGGGACTCATCTCGTCTCGCAAAAC
GTCAGAAGCTGTTGACATTCTCAAGCTGATGTCGACGACGTTCCTTGTGGCTATATGCCAAGCTGTTGATTTGAGACATCTTGAGGAGAATCTGAGACAGACGGTGAAGAACACAGTTTCTCAAGTGG
CGAAGAAAGTGTTAACCACTGGAGTCAACGGTGAGCTGCATCCGTCGCGGTTCTGCGAGAAGGACTTGCTTAAGGTTGTTGATCGCGAGCAAGTGTTCACGTACGTGGATGATCCTTGTCTCGCCAC
GTACCCGTTGATGCAGAAACTGAGACAAGTTATTGTTGATCACGCTTTGTCTAACGGTGAGACTGAGAAGAACGCAGTGACTTCGATCTTTCAAAAGATCGGAGCTTTCGAGGAAGAGCTCAAGATGG
TGCTTCCTAAAGAAGTGGATGCGGCTAGAGAGGCTTACGGTAACGGAACGGCGGCGATTCCGAACAGGATTAAGGAATGTCGGTCTTATCCGTTGTATAAGTTCGTGAGGGAAGAGCTTGGAACGAA
GTTGTTGACCGGAGAAAAGGTTGTGTCTCCGGGAGAGGAGTTTGATAAGGTGTTCACTGCAATGTGTGAAGGTAAGATCATTGATCCATTGATGGAGTGCCTCAAGGAATGGAACGGAGCTCCGATTC
CTATATGCTAA

>BnaPAL2-1_C04p025700.1
MDQINGSVHQNGKTEAMLLCGVVEKTKVTVAADPLNWGAAAEQMKGSHLDEVKRMVEDYRKPVVNLGGETLTIGQVAAISNVGGGVKVELAEASRAGVKASSDWVMESMGKGTDSYGVTTGFGATSHRRT
KNGAALQTELIRFLNAGIFGNTKETCHTLPESATRAAMLVRVNTLLQGYSGIRFEILEAITSLLNHNISPSLPLRGTITASGDLVPLSYIAGLLTGRPNSKATGPNGESLTGEEAFKKAGITSGFFDLQPKEGLALVN
GTAVGSGMASMVLFEANVQSVLAEVLSAIFAEVMSGKPEFTDHLTHRLKHHPGQIEAAAIMEHILDGSSYMKLAQKLHEMDPLQKPKQDRYALRTSPQWLGPQIEVIRHATKSIEREINSVNDNPLIDVSRNKAIH
GGNFQGTPIGVSMDNTRLAVAAIGKLMFAQFSELVNDFYNNGLPSNLTASNNPSLDYGFKGAEIAMASYCSELQYLANPVTSHVQSAEQHNQDVNSLGLISSRKTSEAVDILRLMSTTFLVAICQAVDLRHLEEN
LRQTVKNTVSQVAKKVLTTGVNGELHPSRFCEKDLLKVVDREQVFTYVDDPCSATYPLMQKLRQVIVDHALSNGEIEKNAVTSIFQKIGAFEEELKMVLPKEVDATREAYANGTAAIPNRIKECRSYPLYKFVRE
ELGTKLLTGEKVVSPGEEFDKVFTAMCEGKIIDPLMDCLKEWNGAPIPIC*

>BnaPAL2-1_C04p025700.1_BnaEXP
ATGGATCAGATCAACGGATCAGTTCACCAAAACGGCAAAACCGAAGCGATGTTGTTGTGCGGCGTAGTAGAGAAGACTAAAGTGACGGTGGCGGCGGATCCGTTAAATTGGGGTGCTGCGGCGGAG
CAGATGAAAGGGAGTCATTTAGATGAGGTGAAGAGGATGGTTGAGGATTACCGTAAACCGGTGGTGAATCTCGGCGGAGAAACACTGACGATCGGACAAGTCGCGGCGATCTCGAACGTAGGCGGT
GGCGTTAAGGTTGAGCTAGCGGAGGCTTCGAGAGCTGGCGTGAAAGCTAGCAGCGATTGGGTTATGGAGAGTATGGGTAAAGGTACTGACAGTTACGGTGTAACCACTGGGTTTGGAGCTACCTCTC
ACCGGAGAACCAAAAACGGCGCCGCATTGCAAACCGAACTCATCAGGTTTTTGAACGCCGGAATCTTCGGGAACACGAAGGAGACATGTCACACGCTACCGGAATCCGCCACGAGAGCCGCCATGC
TCGTCCGAGTCAACACACTCCTCCAAGGATACTCCGGGATCCGATTCGAAATCCTCGAAGCGATCACCAGCCTCCTCAACCACAACATCTCTCCGTCTCTCCCCCTCCGCGGAACCATAACCGCCTCC
GGCGATCTCGTCCCCCTCTCCTACATCGCAGGTCTCCTCACCGGCCGTCCGAACTCCAAAGCCACCGGTCCCAACGGTGAATCCCTAACCGGCGAAGAAGCCTTCAAGAAAGCCGGAATCACTTCCG
GATTCTTCGATCTACAGCCTAAGGAAGGCTTAGCGCTCGTCAACGGCACGGCGGTTGGATCCGGCATGGCGTCGATGGTTCTCTTCGAAGCGAATGTTCAATCGGTCCTCGCCGAGGTTTTATCCGC
GATCTTCGCGGAGGTGATGAGCGGGAAGCCGGAGTTCACCGATCATTTGACTCACCGACTAAAACATCACCCCGGACAGATCGAAGCGGCGGCGATCATGGAGCACATCCTCGACGGAAGCTCGTA
CATGAAGCTAGCGCAAAAGCTTCACGAGATGGATCCGTTACAAAAACCGAAACAAGACCGTTACGCGCTCCGCACGTCTCCTCAATGGCTCGGCCCTCAGATCGAAGTGATCCGTCACGCCACGAAG
TCAATCGAGCGTGAGATCAACTCAGTTAACGATAATCCGTTAATTGACGTTTCGAGGAACAAAGCGATTCACGGTGGTAACTTCCAGGGGACTCCGATAGGTGTCTCTATGGACAACACACGTTTAGC
GGTTGCAGCTATTGGGAAGCTCATGTTCGCTCAGTTCTCTGAGTTGGTGAACGACTTCTATAACAACGGTCTTCCTTCGAATCTAACAGCTTCCAACAACCCAAGTTTAGATTACGGATTCAAAGGAGC
AGAGATCGCCATGGCTTCTTACTGCTCTGAGCTTCAGTACCTTGCGAATCCAGTAACAAGCCATGTTCAATCAGCTGAACAGCATAACCAAGATGTTAACTCTTTAGGACTCATCTCCTCTCGTAAGAC
GTCAGAAGCTGTTGACATTCTCAGGCTGATGTCTACCACGTTCCTCGTAGCGATTTGTCAAGCTGTTGATTTGAGACATCTTGAGGAGAATCTAAGACAAACCGTGAAGAACACTGTTTCTCAAGTGGC
GAAGAAAGTGTTGACCACTGGAGTCAACGGGGAGCTGCATCCTTCACGGTTCTGTGAGAAAGACTTGCTTAAGGTCGTTGATCGTGAACAAGTGTTTACATACGTTGACGATCCTTGTAGCGCTACTTA
CCCGTTGATGCAGAAGTTAAGACAAGTCATTGTCGATCACGCTTTATCCAATGGTGAGATTGAGAAGAACGCAGTGACTTCGATCTTTCAAAAGATTGGGGCTTTTGAGGAGGAGCTTAAGATGGTGCT
TCCAAAGGAAGTGGATGCGACTAGAGAGGCTTACGCTAATGGAACGGCGGCGATTCCCAACAGGATTAAGGAATGTCGATCTTATCCGCTGTATAAGTTCGTGAGGGAAGAGCTCGGAACGAAGTTG
TTGACCGGAGAAAAGGTTGTCTCTCCGGGAGAGGAGTTTGATAAGGTATTCACTGCAATGTGTGAAGGTAAGATTATTGATCCATTGATGGATTGTCTCAAGGAATGGAACGGAGCTCCCATTCCCATA
TGTTAA

>BnaPAL2-5_C08p028640.1
MDQTNGSVHQNGKIEAMLCGGVEKTKVAVAADPLNWGAAAEQMKGSHLDELKRMVEEYRRPVVNLGGETLTIGQVAAISTAGGGVKVELAEASRAGVKASSDWVMESMGKGTDSYGVTTGFGATSHRRTK
NGTALQTELIRFLNAGIFVNTLLQGYSGIRFEILEAITSFLNHNISPSLPLRGTITASGDLVPLSYIAGLLTGRPNSKATGPNGESLTAEEAFKQAGIASGFFDLQPKEGLALVNGTAVGSGMASMVLFEANVQSVLA
EVLSAIFAEVMSGKPEFTDHLTHRLKHHPGQIEAAAIMEHILDGSSYMKLAQKLHEMDPLQKPKQDRYALRTSPQWLGPQIEVIRHATKSIEREINSVNDNPLIDVSRNKAIHGGNFQGTPIGVSMDNTRLAIASIG

KLMFAQFSELVNDFYNNGLPSNLTASNNPSLDYGFKGAEIAMASYCSELQYLANPVTSHVQSAEQHNQDVNSLGLISSRKTSEAVDILKLMSTTFLVAICQAVDLRHLEENLRQTVKNTVSQVAKKVLTTGVNG<br>ELHPSRFCEKDLLKVVDREQVFTYVDDPCSATYPLMQKLRQVIVDHALSNGETEKNAVTSIFQKIGAFEEELKMVLPKEVDAAREAYGNGTAAIPNRIKECRSYPLYKFVREELGTKLLTGEKVVSPGEEFDKVF<br>TAMCEGKIIDPLMECLKEWNGAPIPIC*
>BnaPAL2-5_C08p028640.1_BnaEXP<br>ATGGATCAGACTAACGGTTCAGTTCACCAGAACGGCAAGATCGAAGCCATGTTGTGCGGTGGAGTAGAGAAGACAAAAGTGGCTGTGGCGGCGGATCCGTTGAACTGGGGTGCAGCAGCGGAACAG<br>ATGAAAGGGAGTCACTTGGATGAGTTGAAGAGGATGGTTGAGGAGTACCGCAGACCGGTGGTGAATCTCGGGGGAGAGACGCTAACAATCGGACAAGTCGCGGCGATCTCCACCGCAGGCGGAGG<br>TGTTAAGGTTGAGCTAGCGGAGGCTTCGAGAGCTGGCGTGAAAGCTAGCAGTGATTGGGTTATGGAAAGTATGGGCAAAGGTACTGACAGTTACGGTGTCACCACCGGGTTTGGAGCGACCTCTCAC<br>CGGAGAACCAAAAACGGCACCGCATTGCAAACGGAACTCATCAGATTTTTGAACGCCGGAATATTCGTCAACACTCTCCTCCAAGGATACTCCGGGATCCGATTCGAAATCCTCGAAGCGATCACCAG<br>CTTCCTCAACCACAACATCTCTCCATCTCTCCCCCTCCGTGGAACCATAACCGCCTCCGGCGATCTCGTCCCCCTCTCCTACATCGCCGGCCTCCTCACCGGCCGTCCGAACTCCAAAGCCACAGGT<br>CCCAACGGCGAATCCCTAACCGCCGAAGAGGCCTTCAAACAAGCCGGAATCGCTTCCGGATTCTTCGATCTACAACCCAAGGAAGGCTTAGCGCTCGTTAACGGCACGGCGGTTGGATCCGGCATG<br>GCGTCGATGGTTCTATTCGAAGCGAACGTTCAATCGGTGTTAGCGGAGGTCTTATCCGCGATCTTCGCGGAGGTGATGAGCGGGAAGCCTGAGTTCACCGATCATCTAACGCACAGGCTGAAGCACC<br>ACCCCGGGCAAATCGAAGCGGCGGCGATCATGGAGCACATCCTCGACGGAAGCTCGTACATGAAGCTAGCGCAGAAGCTTCACGAGATGGATCCGTTACAAAAGCCGAAACAAGACCGTTACGCTC<br>TCCGTACCTCTCCTCAGTGGCTCGGCCCTCAGATCGAAGTGATCCGTCACGCAACGAAGTCGATCGAGCGTGAGATCAACTCCGTTAACGATAATCCGTTGATCGATGTTTCTAGAAACAAAGCGATT<br>CACGGTGGTAACTTCCAGGGGACTCCAATCGGAGTCTCCATGGATAACACTCGTTTAGCGATTGCTTCGATAGGGAAGCTCATGTTCGCTCAATTCTCGGAGCTTGTTAACGATTTCTACAACAACGGT<br>CTTCCTTCGAATCTAACGGCTTCGAACAATCCAAGCTTGGATTACGGATTCAAAGGAGCTGAGATCGCTATGGCTTCGTATTGCTCTGAGCTTCAGTACTTGGCGAATCCAGTTACAAGCCATGTTCAA<br>TCAGCTGAGCAACATAACCAAGATGTGAACTCTTTGGGACTCATCTCGTCTCGCAAAACGTCAGAAGCTGTTGACATCCTCAAGCTTATGTCGACAACGTTCCTTGTGGCTATATGCCAAGCTGTTGAT<br>TTGAGACATCTTGAGGAGAATCTGAGACAAACGGTGAAGAACACAGTTTCTCAAGTGGCGAAGAAAGTTTTAACCACTGGAGTCAACGGTGAGCTGCATCCGTCGCGGTTCTGCGAGAAGGACTTGCT<br>TAAGGTCGTTGATCGCGAGCAAGTGTTCACGTACGTGGATGATCCTTGTAGCGCTACGTACCCGTTGATGCAGAAACTGAGACAAGTTATTGTTGATCATGCTTTGTCTAACGGTGAGACTGAGAAGAA<br>CGCAGTGACTTCGATCTTTCAAAAGATCGGAGCTTTCGAGGAAGAGCTCAAGATGGTGCTCCCTAAAGAAGTGGATGCGGCTAGAGAGGCTTACGGTAACGGAACGGCGGCGATTCCGAACAGGATT<br>AAGGAATGTCGGTCTTACCCGTTGTATAAGTTCGTGAGGGAAGAGCTTGGAACGAAGCTGTTGACCGGAGAAAAGGTTGTGTCTCCGGGAGAGGAGTTTGATAAGGTGTTCACTGCAATGTGTGAAG<br>GTAAGATCATTGATCCGTTGATGGAGTGCCTCAAGGAATGGAACGGAGCTCCGATTCCGATATGCTAA
>BnaPAL2-3_R186_chrC06p000250.1<br>MDQTQNDNIEAMLCGGVEKTNVAVAADPLNWGAAAEQMKGSHLDEVKRMVEEYRRPVVNLGGETLTIGQVAAISTVGNGVKVELAEASRDGVKASSDWVMESMGKGTDSYGVTTGFGATSHRRTKNGAAL<br>QTELIRFLNAGIFVNTLLQGYSGIRFEILEAITSLLNHNISPSLPLRGTITASGDLVPLSYIAGLLTGRPNSKATGPNGESLTAEEAFKQAGITSGFFDLQPKEGLALVNGTAVGSGMASMVLFEANVQSVLAEVLSAI<br>FAEVMSGKPEFTDHLTHRLKHHPGQIEAAAIMEHILDGSSYMKLAQKLHEMDPLQKPKQDRYALRTSPQWLGPQIEVIRYATKSIEREINSVNDNPLIDVSRNKAIHGGNFQGTPIGVSMDNTRLAVAAIGKLMFA<br>QFSELVNDFYNNGLPSNLTASNNPSLDYGFKGAEIAMASYCSELQYLANPVTSHVQSAEQHNQDVNSLGLISSRKTSEAVDILKLMSTTFLVAICQAVDLRHLEENLRQAVKNTVSQVAKKVLTTGVNGEMHPS<br>RFCERDLLKVVDREQVFTYVDDPCSATYPLMQKLRQVIVDQALANGETEKNVETSIFQKIGAFEEELKTVLPKEVDAAREAYGNGNAAIPNRIKECRSYPLYKFVREELGTKLLTGEKVVSPGEEFDKVFTAMCE<br>GKIIDPLMDCLKEWNGAPIPIC*
>R186_chrC06p000250.1_BnaEXP<br>ATGGATCAGACCCAAAACGACAACATCGAAGCTATGTTGTGCGGCGGAGTTGAGAAGACGAACGTTGCTGTGGCGGCAGATCCGTTGAACTGGGGTGCTGCGGCGGAGCAGATGAAAGGGAGCCAT<br>TTGGATGAGGTGAAGAGGATGGTTGAGGAGTACCGTAGACCGGTGGTGAACCTCGGAGGAGAGACGCTGACGATCGGACAAGTGGCGGCGATCTCCACCGTCGGTAACGGCGTTAAGGTTGAGCT<br>AGCGGAGGCTTCGAGAGATGGAGTGAAAGCTAGCAGCGATTGGGTTATGGAGAGTATGGGCAAAGGTACCGACAGTTACGGTGTCACCACCGGGTTTGGAGCTACCTCTCACCGGAGAACGAAAAA<br>CGGAGCTGCATTGCAGACGGAGCTCATCAGATTTTTGAACGCCGGAATATTCGTCAACACACTCCTCCAAGGATACTCCGGGATCCGATTCGAAATCCTCGAAGCGATCACCAGTCTCCTCAACCACA<br>ACATCTCTCCATCTCTCCCCCTACGTGGAACCATAACCGCCTCCGGCGATCTCGTCCCCCTCTCCTACATCGCCGGTCTGCTCACCGGACGCCCCAACTCCAAAGCCACCGGTCCCAACGGCGAATC<br>CCTAACCGCCGAAGAAGCCTTCAAACAAGCCGGAATCACTTCCGGATTCTTCGATCTGCAACCTAAGGAAGGTCTCGCGCTCGTTAACGGCACGGCGGTTGGATCCGGCATGGCGTCGATGGTTCTA<br>TTCGAAGCGAATGTTCAATCCGTTTTAGCGGAGGTTTTATCGGCGATCTTCGCGGAGGTGATGAGCGGGAAGCCTGAGTTCACCGATCATTTGACTCACCGACTCAAACATCACCCCGGACAAATCGA<br>AGCGGCGGCGATCATGGAGCACATCCTCGACGGAAGCTCGTACATGAAGCTAGCTCAAAAGCTTCACGAGATGGATCCGTTACAGAAGCCCAAACAAGACCGTTACGCTCTCCGTACTTCTCCTCAAT<br>GGCTCGGCCCTCAGATCGAAGTGATCCGTTACGCCACGAAATCAATCGAGCGTGAGATCAACTCCGTTAACGATAATCCGTTGATCGACGTTTCTAGGAACAAAGCCATTCACGGTGGTAACTTCCAG<br>GGGACGCCGATTGGAGTTTCGATGGACAACACGCGTTTAGCGGTTGCAGCCATTGGGAAGCTCATGTTTGCTCAGTTCTCTGAGCTGGTCAACGATTTCTACAATAATGGCCTTCCTTCGAATCTCAC<br>AGCTTCTAACAATCCAAGTTTGGATTACGGTTTCAAAGGAGCAGAGATCGCTATGGCTTCTTACTGCTCTGAGCTTCAGTACTTGGCGAATCCAGTTACTAGCCATGTTCAATCAGCTGAACAGCATAAC<br>CAAGACGTTAACTCTTTAGGACTCATCTCATCTCGTAAGACCTCAGAAGCTGTTGACATTCTCAAGCTAATGTCTACCACGTTCCTTGTTGCTATATGCCAAGCTGTTGACTTGAGACATCTTGAGGAGA<br>ATCTGAGACAGGCGGTGAAGAACACAGTTTCTCAAGTGGCGAAGAAAGTGTTGACTACTGGAGTCAACGGGGAGATGCATCCTTCACGGTTCTGTGAGAGAGACTTGCTTAAGGTCGTTGACCGTGA<br>GCAAGTGTTCACGTACGTGGATGATCCTTGTAGCGCAACTTACCCACTGATGCAGAAGCTAAGACAAGTCATTGTTGATCAAGCTTTAGCCAACGGTGAGACTGAGAAGAACGTAGAGACTTCAATTTT<br>TCAAAAGATTGGAGCTTTTGAGGAGGAGCTTAAGACGGTTCTTCCTAAGGAAGTGGATGCGGCTAGAGAGGCTTACGGTAATGGAAACGCAGCGATTCCGAACAGGATTAAGGAGTGTCGGTCTTATC<br>CGTTGTATAAGTTCGTGAGGGAGGAGCTTGGAACGAAGTTGTTGACGGGAGAAAAGGTTGTGTCTCCGGGAGAGGAGTTTGATAAGGTGTTCACTGCTATGTGTGAAGGTAAGATTATTGATCCACTG<br>ATGGATTGTCTCAAGGAATGGAACGGAGCTCCGATTCCGATATGCTAA

>BnaPAL2-4_A07p016620.1
MDQTQNDNIETMLCGGVEKTNVAVAADPLNWGAAAEQMKGSHLDEVKRMVEEYRRPVVNLGGETLTIGQVAAISTVGNGVKVELAEASRAGVKASSDWVMESMGKGTDSYGVTTGFGATSHRRTKNGVAL
QTELIRFLNAGIFVNTLLQGYSGIRFEILEAITSLLNHNISPSLPLRGTITASGDLVPLSYIAGLLTGRPNSKATAPNGESLTAEEAFKQAGIASGFFDLQPKEGLALVNGTAVGSGMASMVLFEANVQSVLAEVLSAI
FAEVMSGKPEFTDHLTHRLKHHPGQIEAAAIMEHILKGSSYMKLAQKVHEMDPLQKPKQDRYALRTSPQWLGPQIEVIRYATRSIEREINSVNDNPLIDVSRNKAIHGGNFQGTPIGVSMDNTRLAVAAIGKLMF
AQFSELVNDFYNNGLPSNLTASNNPSLDYGFKGAEIAMASYCSELQYLANPVTSHVQSAEQHNQDVNSLGLISSRKTSEAVDILKLMSTTFLVAICQAVDLRHLEENLRQAVKNTVSLVAKKVLTTGVNGEMHP
SRFCERDLLKVVDREQVFTYVDDPCSASYPLMQKLRQVIVDHALANGETEKNVETSIFEKIGAFEEELKTVLPKEVDAAREAYGNGNAAIPNRIKECRSYPLYKFVREELGTKLLTGEKVVSPGEEFDKVFIAMCE
GKIIDPLMDCLKEWNGAPIPIC*

>BnaPAL2-4_A07p016620.1_BnaEXP
ATGGATCAGACACAAAACGACAACATCGAAACTATGTTGTGCGGCGGAGTTGAGAAGACGAACGTGGCTGTGGCGGCAGATCCGTTGAACTGGGGTGCTGCGGCGGAGCAGATGAAAGGGAGCCAC
TTGGATGAGGTGAAGAGGATGGTTGAGGAGTACCGTAGACCGGTGGTGAACCTCGGAGGAGAGACGCTAACGATCGGACAAGTGGCGGCGATCTCCACCGTCGGTAACGGCGTTAAGGTTGAGCTA
GCGGAGGCTTCGAGAGCGGGAGTGAAAGCTAGCAGCGATTGGGTTATGGAGAGTATGGGCAAAGGTACTGACAGTTACGGTGTCACCACCGGGTTTGGAGCTACCTCTCACCGTAGAACGAAAAAC
GGGGTCGCATTGCAGACAGAACTCATCAGATTTTTGAACGCCGGAATATTCGTCAACACACTCCTCCAAGGATACTCCGGGATCCGATTCGAAATCCTCGAAGCGATCACCAGTCTCCTCAACCACAA
CATCTCCCCATCTCTCCCCCTCCGTGGAACCATAACCGCCTCCGGCGACCTCGTCCCCCTCTCCTACATCGCCGGTCTCCTCACCGGACGCCCCAACTCCAAAGCCACCGCTCCCAACGGCGAATCC
CTAACCGCCGAAGAAGCCTTCAAACAAGCCGGAATCGCTTCCGGATTCTTCGATCTTCAACCCAAGGAAGGTCTCGCGCTCGTTAACGGCACGGCGGTTGGATCCGGCATGGCCTCGATGGTTCTAT
TCGAAGCGAACGTTCAGTCCGTTTTAGCGGAGGTTTTATCGGCGATCTTCGCGGAGGTGATGAGCGGGAAGCCTGAGTTCACCGATCATTTGACTCACCGACTCAAACATCACCCCGGACAAATCGA
AGCGGCGGCGATCATGGAGCACATCCTCAAGGGAAGCTCGTACATGAAGCTAGCTCAAAAGGTTCACGAGATGGATCCGTTACAGAAGCCCAAACAAGACCGTTACGCTCTCCGTACTTCTCCTCAAT
GGCTCGGCCCTCAGATCGAAGTGATCCGTTACGCGACGAGATCAATCGAGCGTGAGATCAACTCCGTTAACGATAATCCGTTGATCGACGTTTCTAGGAACAAAGCGATTCACGGTGGTAACTTCCAG
GGGACGCCGATTGGAGTTTCGATGGACAACACGCGTTTAGCGGTTGCGGCCATTGGGAAGCTCATGTTTGCTCAGTTCTCGGAGCTTGTTAACGATTTCTACAACAATGGTTTACCTTCGAACTTAACA
GCTTCGAATAATCCAAGTTTGGATTACGGTTTCAAAGGAGCTGAGATCGCTATGGCTTCTTACTGCTCTGAGCTTCAGTACTTAGCGAATCCAGTAACTAGCCATGTTCAATCAGCTGAGCAGCATAAC
CAAGACGTTAACTCTTTAGGACTCATCTCATCTCGTAAGACCTCAGAAGCTGTTGACATTCTCAAGCTGATGTCTACGACGTTCCTTGTTGCTATATGCCAAGCTGTTGACTTGAGACATCTTGAGGAGA
ATCTGAGACAGGCGGTGAAGAACACAGTTTCTCTAGTGGCGAAGAAAGTGTTGACCACTGGAGTCAACGGGGAGATGCATCCGTCACGGTTCTGTGAGAGAGACTTGCTTAAGGTCGTTGACCGTGA
GCAAGTGTTCACGTACGTGGATGATCCTTGCAGCGCAAGTTACCCGTTGATGCAGAAGCTAAGACAAGTCATTGTTGATCACGCTTTAGCCAACGGTGAGACTGAGAAGAATGTGGAGACTTCAATCT
TTGAAAAGATTGGAGCTTTTGAGGAGGAGCTTAAGACGGTTCTTCCTAAGGAAGTGGATGCGGCTAGAGAGGCTTACGGTAATGGAAACGCAGCGATTCCGAACAGGATTAAGGAGTGTCGGTCTTAT
CCGTTGTATAAGTTCGTGAGGGAGGAGCTTGGAACGAAGTTGTTGACCGGAGAAAAGGTTGTGTCTCCGGGAGAGGAGTTTGATAAGGTGTTCATTGCTATGTGTGAAGGTAAGATTATTGATCCACT
GATGGATTGTCTCAAGGAATGGAACGGAGCTCCGATTCCGATATGCTAA

>BnaPAL4-2_A05p031299.1
MDLCKQNNNHIVAVSADPLNWNAAAEALKGSHLEEVKRMVEDYRKGAVRLGGETLTIGQVAAVASGGVTVELAEEARAGVKASSDWVMESMNRGTDSYGVTTGFGATSHRRTKQGGALQKELIRFLNAGIF
GAGVGDTSLTLPKSATRAAMLVRVNTLLQGYSGIRFEILEAITKLLNNEITPCIPLRGSITASGDLVPLSYIAGLLTGRPNSKAVGPAGETLTASDAFKLAGVPSFFELQPKEGLALVNGTGVGSGLASMVLFETNV
LAVLSEVMSAMFAEVMQGKPEFTDHLTHKLKHHPGQIEAAAIMEHILHGSSYVKEAQQLHELDPLQKPKQDRYALRTSPQWLGPQIEVIRAATKMIEREINSVNDNPLIDVSRNKALHGGNFQGTPIGVAMDNT
RLAIASIGKLMFAQFSELVNDFYNNGLPSNLSGGRNPSLDYGFKGAEIAMASYCSELQFLANPVTNHVQSAEQHNQDVNSLGLISSRKTEEAVDILKLMSTTYLVALCQAVDLRHIEENLKKAVKAAVSQVAKRV
LTVGVNGELHPSRFTERDVLQVVDREHVFSYADDPCSFAYPLMQRLRHVLVDHALEDPDREANVSTSVFQKIEAFEAELKVVLPKEVERVRVEYEGGCSVVGNRIKECRSYPLYRFVREELETELLSGESVRS
PGEEFDKVFSAICDGKVIDPLLECLKEWNGAPVPIC

>BnaPAL4-2_A05p031299.1_BnaEXP
atggacttgtgcaaacaaaacaacaaccacatcgtcgctgtttccgccgatccgttgaactggaacgcggcggcggaagctttgaaagggagccacttggaggaggtgaaacgtatggtggaagattacagaaaaggtgcggtgcggttaggaggagagacgctgacgattggtcaagttg
cagccgtggctagcggaggagtgacggtggagctggcggaggaggctcgtgccggagttaaggcgagtagcgactgggtgatggagagcatgaaccgtggcacggacagttatggagtcaccacagggtttggtgcaacgtcccatagaagaactaaacaaggcggtgcacttcaaaa
ggagcttattaggttcttgaacgccggaatattcggtgccggcgtcggagacacgtcactcacgctacctaagtcggcaactagagcagctatgctcgtccgtgtcaacactctcctccaaggctactctggaatacgctttgagattcttgaagccattacaaagcttcttaacaacgaaatcactcc
atgcatccctctccgtggcagcatcaccgcatccggcgaccttgttcctctcttacatcgccggactgctcaccggccgtcccaattcaaaagccgtgggtcccgccggtgagactctcactgcctccgatgcctttaagctagccggagtaccgtcctttttgagctgcagccaaaggaaggact
ggctcttgtgaacgggacaggggtcggatcgggtctggcttcgatggttctgtttgagaccaatgttttggcggttttgtctgaagttatgtctgcgatgttcgcggaggtcatgcaagggaagccggagtttactgatcatctaacgcataagctcaagcaccatcctggtcagattgaagccgctgcga
ttatggaacatatattacatggaagctcttacgttaaagaagctcagcagcttcatgaactggatccgcttcaaaaacctaaacaagatcggtacgcgctaaggacgtcaccacagtggctaggaccacaaatcgaagtgatcagagcagcgaccaagatgatagagcgcgagatcaactcg
gtcaacgacaaccctctcatcgacgtctccagaaacaaagcactacacggcggtaacttccaaggcacgcctatcggtgtcgccatggacaacacccgcttagccatcgcttccatcggcaaactcatgttcgcgcagttctccgagctcgtgaacgacttctacaacaacggcttgccttcaaa
cctatccggcgggagaaaccctagcctcgactacggtttcaaaggcgcggagatcgccatggcctcttactgctccgagctccagttcttggctaaccccgtgacgaaccacgtccagagcgccgagcagcataaccaagacgttaactcgctagggctgatctcgagtcggaagacagaag
aagctgttgatatcctcaagctgatgtccacgacctacttggttgccttatgccaagccgttgatctgcgtcatatcgaagagaatctcaagaaagctgttaaagccgcggtgagccaggtggcgaaacgggtgttaacggttggtgttaacggcgagctgcatccgtctaggttcacggagcgtgat
gtgctccaagtggttgatagagagcacgtgttctcatacgcggacgatccttgcagctttgcttatccgttgatgcagaggcttaggcacgttcttgtggaccacgctttggaggatccggaccgcgaggctaatgtttcgacgtcggtttttcagaagatagaagcgtttgaggcggagctgaaggtgg
ttttacctaaggaagtggagcgtgttagggtggagtatgagggagggtgttcggttgtgggtaaccggattaaggaatgtcggtcttatccgttgtaccggtttgtgagggaggagctggagactgagctgttgagtggagagagtgttaggtcgccgggtgaggagtttgataaagtgttctcggcga
tttgtgatgggaaggttattgatcctttgttggagtgtctcaaggagtggaacggagctccggttccgatctgttga

>BnaPAL4-1_C05p041260.1

MELCEQNNNHVVAVSADPLNWNAAAEALKGSHLEEVKRMVEDYRKGAVRLGGETLTIGQVAAVASGGVMVELAEEARAGVKASSDWVMESMNRGTDSYGVTTGFGATSHRRTKQGGALQKELIRFLNAGI
FGAGAGDSSLTLPKSATRAAMLVRVNTLLQGYSGIRFEILEAITKLLNNEITPCIPLRGSITASGDLVPLSYIAGLLTGRPNSKAVGPAGETLTASDAFKLAGVPSFFELQPKEGLALVNGTGVGSGLASMVLFEAN
VLAVLSEVMSAMFAEVMQGKPEFTDHLTHKLKHHPGQIEAAAIMEHILHGSSYVKEAQQLHELDPLQKPKQDRYALRTSPQWLGPQIEVIRAATKMIEREINSVNDNPLIDVSRNKALHGGNFQGTPIGVAMDNT
RLAIASIGKLMFAQFSELVNDFYNNGLPSNLSGGRNPSLDYGFKGAEIAMASYCSELQFLANPVTNHVQSAEQHNQDVNSLGLISSRKTEEAVEILKLMSTTYLVALCQAVDLRHIEENLKKAVKAAVSQVAKRV
LTVGVNGELHPSRFTERDVLQVVDREHVFSYADDPCSFAYPLMQRLRHVLVDHALEDPDREADASASVFQKIGAFETELKVVLPKEVERVRGEYEGGRSVVGNRIKECRSYPLYRFVREELETELLSGESVRS
PGEVFDKVFSAICDGKVIDPLLECLKEWNGAPVPIC*

>BnaPAL4-1_C05p041260.1_BnaEXP
ATGGAGTTATGCGAACAAAACAACAACCACGTCGTCGCTGTCTCCGCCGATCCGTTGAACTGGAACGCGGCGGCGGAAGCTTTGAAAGGGAGCCACTTGGAGGAGGTGAAACGTATGGTGGAAGAT
TACAGAAAAGGGGCGGTGCGGTTAGGAGGAGAGACGCTGACGATTGGTCAAGTTGCCGCCGTTGCTAGCGGAGGAGTGATGGTGGAGCTGGCGGAGGAGGCTCGTGCCGGAGTTAAGGCGAGTA
GCGACTGGGTGATGGAGAGCATGAACCGTGGCACGGACAGTTATGGAGTCACCACGGGGTTTGGTGCAACGTCCCATAGAAGAACTAAACAAGGCGGTGCACTTCAAAAGGAGCTTATTAGGTTCTT
GAACGCCGGGATATTCGGTGCCGGCGCCGGAGACTCGTCACTCACGCTACCTAAGTCGGCGACTAGAGCAGCTATGCTCGTCCGTGTCAACACTCTCCTCCAAGGCTACTCTGGAATACGTTTTGAG
ATTCTTGAAGCCATTACAAAGCTTCTTAACAACGAAATCACTCCATGCATCCCTCTCCGTGGCAGCATCACTGCATCCGGCGACCTTGTTCCCCTCTCTTACATCGCCGGACTACTCACCGGCCGTCCC
AATTCAAAAGCCGTGGGTCCCGCCGGTGAGACTCTCACTGCCTCTGATGCCTTTAAGCTAGCCGGAGTACCGTCCTTTTTCGAGCTGCAGCCAAAGGAAGGACTGGCTCTTGTGAACGGGACAGGGG
TCGGATCGGGTCTGGCCTCGATGGTTCTGTTTGAGGCCAACGTTTTGGCGGTTTTGTCTGAAGTTATGTCTGCCATGTTCGCGGAGGTTATGCAAGGCAAACCGGAGTTTACTGATCATCTAACGCAT
AAGCTCAAGCACCATCCTGGTCAGATTGAAGCCGCTGCGATTATGGAACATATATTACATGGAAGCTCTTACGTTAAAGAAGCTCAACAGCTCCATGAACTGGATCCGCTTCAAAAACCTAAACAAGAT
CGGTACGCGCTAAGGACGTCACCACAATGGCTAGGACCACAAATCGAAGTGATCAGAGCAGCGACCAAGATGATAGAGCGTGAGATCAACTCGGTCAACGACAACCCTCTCATAGACGTCTCCAGAA
ACAAAGCACTACACGGCGGTAACTTCCAAGGCACGCCTATCGGTGTTGCCATGGACAACACCCGCTTAGCCATCGCTTCCATCGGCAAACTCATGTTCGCGCAGTTCTCCGAGCTCGTGAACGACTT
CTACAACAACGGCTTGCCTTCAAACCTATCCGGCGGGAGAAACCCTAGCCTCGACTACGGTTTCAAAGGCGCGGAGATAGCCATGGCCTCTTACTGCTCCGAGCTCCAGTTCTTGGCTAACCCCGTG
ACTAACCACGTCCAGAGCGCCGAGCAGCATAACCAAGACGTTAACTCGCTAGGGCTGATCTCGAGCCGGAAGACAGAAGAAGCTGTTGAGATCCTCAAGCTGATGTCCACGACCTACTTGGTTGCGC
TCTGCCAAGCCGTTGATCTGCGTCATATCGAAGAGAATCTCAAGAAAGCGGTTAAAGCCGCTGTGAGCCAGGTGGCGAAACGGGTGTTAACGGTTGGTGTTAACGGCGAGCTGCATCCGTCGAGGTT
CACGGAGCGGGATGTGCTCCAAGTGGTTGATAGAGAGCACGTGTTCTCCTACGCGGACGATCCTTGCAGCTTTGCTTACCCGTTGATGCAGAGGCTTAGGCACGTTCTTGTGGACCACGCTTTAGAG
GATCCGGACCGCGAGGCTGATGCTTCGGCGTCGGTTTTTCAGAAGATAGGAGCGTTTGAGACGGAGCTGAAGGTGGTTTTACCTAAGGAGGTGGAGCGTGTTAGGGGTGAGTATGAGGGAGGGCG
GTCGGTTGTGGGTAACCGGATTAAGGAATGTCGGTCTTATCCGTTGTACCGGTTTGTGAGGGAGGAGCTGGAGACTGAGCTGTTGAGTGGAGAGAGTGTTAGGTCGCCCGGTGAAGTGTTTGATAAA
GTGTTCTCGGCGATTTGTGATGGGAAGGTTATTGATCCTTTGTTGGAGTGTCTCAAGGAGTGGAACGGAGCTCCGGTTCCGATCTGTTGA

>BnaPAL3-2_A04p012590.1
MEFCQPNKNNGSASSDPLNWNVAAEALKGSHVEDVKKMVEDYRKGTVRLGGETLTIGQVAAVASKGTTVELLEEARAGVKASSEWVMESINRGTDTYGITTGFGSSSRRRTNQGAALQKELIRYLNTGIFAT
GDEDDVLSNILPRPATRAAMLIRVNTLLQGYSGIRFEILEAITKFLNHKITLRLPLRGTITASGDLVPLSYIAGLLTGRPNSRSVGPSDEILTALEAFKLAGISSPFELRPKEGLALVNGTAVGSAMASIVLYEANVLAV
FSEVASAMFVEVMHGKPEFTDHLVHKLKHHPGQIEAAAIMEHILDGSSFVKEAIRLHEIDPLQKPKQDRYALRTSPQWLGPQIEVIRAATKMIEREINSVNDNPLIDVLRNKAIHGGNFQGTPIGVAMDNARLAIGS
IGKLMFAQFTELVNDFYNNGLPSNLSGGRNPSLDYGFKGAEVPMAAYCSELQFLANPVTNHVQSTEQHNQDVNSLGLVSSHKTAEAVNILKLMSATYLVALCQAYDLRHLEDNLKETIKAVVNQTAERHVFTLS
KPFLEQNILGVIDREYVFSYVYDLSSLTNPLMQKLRSILFDHALAEPEHETDSGFRKIGTFETELKSLLHNEVERVWTEYEKGNFVVANRIKECRSYPLYRFVREELETRLLTGGSVRTPGEDFDEVFKAISKGKLI
DPLFECLKEWNGAPIPIS*

>BnaPAL3-2_A04p012590.1_BnaEXP
ATGGAATTTTGTCAACCAAACAAAAACAACGGAAGTGCGTCTAGTGATCCACTTAATTGGAACGTAGCGGCCGAGGCTTTGAAAGGGAGCCACGTGGAGGATGTGAAGAAGATGGTGGAGGATTATA
GGAAAGGAACGGTGCGGCTCGGTGGAGAGACGCTGACTATCGGTCAGGTTGCGGCCGTTGCGAGCAAAGGGACGACGGTGGAGCTTTTGGAGGAGGCTCGTGCCGGCGTGAAGGCTAGTAGTGA
GTGGGTGATGGAAAGCATTAACCGTGGCACGGACACTTATGGAATCACCACTGGATTTGGTTCTTCTTCTCGTAGGAGGACCAATCAAGGTGCTGCTCTTCAAAAAGAGCTTATTAGGTACTTGAACAC
CGGAATATTCGCTACAGGCGACGAAGATGACGTTTTGTCAAACATTCTTCCTCGTCCGGCAACAAGAGCGGCGATGCTCATCCGTGTCAACACACTCCTCCAAGGCTACTCCGGTATACGCTTCGAAA
TCCTTGAAGCCATCACAAAGTTCCTCAACCACAAAATCACACTGCGCCTCCCTCTCCGAGGCACCATCACCGCCTCTGGTGACCTCGTTCCTCTATCGTACATCGCCGGTCTCCTCACCGGACGGCCC
AACTCCCGATCCGTGGGTCCATCCGATGAGATCCTTACTGCCTTAGAGGCCTTCAAGCTAGCTGGAATATCTTCTCCTTTTGAGCTCCGGCCTAAAGAAGGGCTTGCGCTCGTGAACGGCACCGCGG
TTGGGTCCGCTATGGCCTCAATAGTACTATACGAGGCCAACGTTCTGGCAGTTTTTTCTGAAGTTGCTTCCGCCATGTTTGTTGAGGTTATGCATGGGAAACCCGAGTTTACCGATCATCTTGTGCATA
AACTCAAGCACCATCCTGGTCAGATCGAAGCCGCGGCAATCATGGAACATATCTTAGACGGAAGCTCTTTTGTCAAAGAAGCTATACGTCTCCATGAGATTGATCCGCTCCAAAAACCTAAACAAGATC
GCTACGCTCTGCGAACATCGCCACAGTGGCTTGGACCGCAGATTGAGGTGATCAGAGCCGCGACGAAAATGATTGAACGAGAGATAAACTCTGTCAACGATAACCCTTTAATCGATGTGCTGCGAAAC
AAAGCTATCCACGGTGGGAATTTCCAGGGGACACCAATAGGTGTAGCCATGGACAACGCTCGTCTGGCCATTGGTTCCATCGGGAAACTAATGTTTGCGCAATTCACGGAACTCGTCAATGATTTCTA
TAACAACGGTCTACCTTCGAATCTATCCGGTGGGAGAAACCCTAGCCTTGACTACGGGTTCAAAGGCGCGGAAGTCCCCATGGCTGCTTATTGTTCCGAGCTTCAGTTCTTGGCTAACCCTGTGACAA
ACCATGTCCAAAGCACTGAGCAACATAATCAAGATGTTAACTCTCTTGGTTTGGTCTCCAGCCACAAGACTGCAGAAGCTGTGAATATCCTCAAGCTTATGTCAGCTACTTACTTAGTAGCTTTATGCCA
AGCCTATGATCTAAGACATCTTGAAGATAATCTCAAGGAAACGATTAAGGCGGTTGTGAACCAAACCGCGGAAAGACACGTATTCACGCTCAGCAAACCGTTCCTCGAACAGAACATCCTCGGAGTTAT
CGACCGCGAATATGTCTTTTCCTATGTTTATGACCTAAGCAGCCTCACTAACCCTCTAATGCAGAAGCTGAGAAGCATTCTTTTCGACCATGCTTTAGCTGAACCGGAACATGAGACGGATTCGGGTTT
TCGAAAAATAGGAACATTCGAAACAGAGCTGAAATCTCTTCTACATAACGAAGTTGAAAGAGTGTGGACCGAGTATGAAAAGGGTAACTTTGTTGTGGCTAACCGGATCAAAGAGTGTAGATCGTATCC

GTTGTACCGGTTTGTACGGGAGGAGCTAGAGACGAGGCTACTAACCGGAGGGAGTGTCCGGACACCAGGTGAAGATTTCGACGAAGTTTTTAAAGCCATCTCTAAGGGAAAACTCATAGATCCTCTGT<br>TTGAATGCCTCAAGGAATGGAACGGAGCTCCAATCCCAATCTCCTAA
>BnaPAL3-1_C04p034430.1<br>MLIRVNTLLQGYSGIRFEILEAITKFLNHKITPRLPLRGTITASGDLVPLSYIAVLLTGRPNSRSVGPSGEILAALEAFKLAGISSPFELRPKEGLALVNGTAVGSAMASIVLYEANVLAVFSEVASAMFAEVMHGKPE<br>FSDHLVHKLKHHPGQMEAAAITEHILDGSSFVKEAIRLHEIDPLQKPKQDRYALRTSPQWLGPQIEVIRAATKMIEREINSVNDNPLIDVSRNKAIHGGNFQGTPIGVAMDNARLAIGSIGKLMFAQFTELVNDFYN<br>NGLPSNLSGGRNPSLDYGFKGAEVPMAAYYSELQFLANPVTNHVQSTEQHNQDVNSLGLISSHKTAEAVNILKLMSATYLVALCQAYDLRHLEDNLKETIKAVVNQTAEKHAFTLSKPFIEQNILGVIDREYVFSY<br>VYDLSSLANPLMQKLRSVLFDHALAEPEHETDSGFRKIGTFETELKSLLPNEVERVWTEYENGNFVVANRIKECRSYPLSRFVREELETRLLTGGSVRTPGEDFDEVFKAISKGKLIDPLFECLKEWNGAPIPIS*
>BnaPAL3-1_C04p034430.1_BnaEXP<br>ATGCTCATCCGTGTCAACACACTCCTCCAAGGCTACTCCGGTATACGCTTCGAAATCCTTGAAGCCATCACAAAGTTCCTCAACCACAAAATCACACCGCGCCTCCCTCTCCGAGGCACCATCACCGC<br>CTCTGGTGACCTCGTTCCTCTATCGTACATCGCCGTTCTCCTCACCGGACGGCCCAACTCCCGATCCGTGGGTCCATCCGGTGAGATCCTTGCCGCCTTAGAGGCCTTCAAGCTAGCTGGAATATCTT<br>CTCCTTTTGAGCTCCGGCCTAAAGAAGGGCTTGCGCTCGTGAACGGCACCGCGGTTGGGTCTGCTATGGCCTCAATAGTGCTATACGAGGCCAACGTTTTGGCAGTGTTCTCGGAAGTTGCTTCTGC<br>CATGTTTGCCGAGGTTATGCATGGGAAACCCGAGTTTTCCGATCATCTTGTGCATAAACTCAAGCACCATCCTGGTCAGATGGAAGCCGCGGCAATCACGGAACATATCTTAGACGGAAGCTCTTTTGT<br>CAAAGAAGCTATACGTCTCCACGAGATTGATCCGCTCCAAAAACCTAAACAAGATCGCTACGCTCTGCGAACATCGCCACAGTGGCTTGGACCTCAGATTGAGGTGATCCGAGCCGCGACGAAAATGA<br>TCGAACGAGAGATAAACTCTGTCAACGATAACCCCTTAATCGATGTGTCGCGAAATAAAGCTATCCACGGTGGGAATTTCCAAGGGACACCAATAGGTGTCGCCATGGACAACGCTCGTCTAGCCATT<br>GGTTCCATCGGGAAACTAATGTTTGCGCAGTTCACGGAACTCGTCAATGATTTCTACAACAACGGTCTACCTTCGAATCTATCCGGTGGGAGAAACCCTAGCCTTGACTACGGGTTTAAAGGCGCGGA<br>AGTCCCCATGGCTGCTTATTATTCCGAGCTTCAGTTCTTGGCTAACCCTGTGACAAACCATGTCCAAAGCACTGAACAACATAATCAAGATGTTAACTCTCTTGGTTTGATCTCCAGCCACAAAACTGCC<br>GAAGCTGTGAATATCCTCAAGCTTATGTCAGCTACTTACTTAGTAGCTTTATGCCAAGCCTATGATCTAAGACATCTTGAAGACAATCTCAAGGAAACGATTAAAGCGGTTGTGAACCAAACCGCGGAAA<br>AACACGCATTCACTCTCAGCAAACCGTTCATCGAACAGAACATCCTCGGAGTTATCGACCGCGAATATGTCTTTTCCTATGTTTATGACCTAAGTAGCCTCGCTAACCCTCTAATGCAGAAGCTGAGAA<br>GCGTTCTTTTCGACCATGCTTTAGCTGAACCGGAGCATGAGACGGATTCGGGTTTTCGAAAAATAGGAACGTTCGAAACAGAGCTGAAATCTCTTCTACCTAACGAAGTTGAAAGAGTGTGGACCGAG<br>TACGAAAATGGTAACTTTGTTGTGGCCAACCGAATCAAAGAGTGTAGATCGTATCCGTTGTCCCGGTTTGTACGGGAGGAGCTAGAGACGAGGCTACTAACCGGAGGGAGTGTCCGGACACCAGGAG<br>AAGATTTTGACGAAGTTTTTAAAGCCATCTCTAAGGGAAAACTCATAGATCCTCTGTTTGAATGTCTCAAAGAATGGAACGGAGCTCCAATTCCAATCTCCTAA

Supplementary Table 7: Organ-specific expression of *B. napus* Express 617 *PAL* candidate genes. The median transcripts per millions (TPMs) of each *PAL* candidate gene per organ is listed. Paired-end RNA-Seq data generated in this study derived from seeds (35 DAF) of Express 617 (E617) and SGDH14 (S14) are marked with an asterisk. Publicly available paired-end *B. napus* RNA-Seq data sets were used for the remaining organs. The number of analysed data sets per organ is stated via (n = X). The colour gradient from white to blue indicates the expression strength with dark blue symbolising high expression. DAF, days after flowering; DAP, days after pollination; SAM, shoot apical meristem.

	PAL4-1 C05p041260.1	*PAL4-2* A05p031299.1	*PAL3-1* C04p034430.1	*PAL3-2* A04p012590.1	*PAL2-1* C04p025700.1	*PAL2-2* A04p005140.1	*PAL2-3* R186_chrC06p000250.1	*PAL2-4* A07p016620.1	*PAL2-5* C08p028640.1	*PAL2-6* A09p036110.1	*PAL1-1* C04p009110.1	*PAL1-2* A05p007980.1	*PAL1-3* C04p046000.1	*PAL1-4* A04p023010.1
SAM (n=16)	1.3	8.4	0.0	0.0	2.3	0.6	0.0	0.0	0.0	2.2	4.2	1.5	8.7	0.1
Anther prophase 1 (n=12)	0.1	0.8	0.0	0.0	3.1	2.4	0.0	0.0	0.0	3.7	0.1	0.0	0.1	0.2
Anther bolting (n=6)	0.7	54.0	0.0	0.1	3.0	1.8	0.0	0.0	0.0	4.5	27.9	14.8	19.9	0.0
Anther flowering (n=4)	0.0	70.0	0.0	0.0	9.0	5.8	0.0	0.0	0.0	13.1	33.1	31.6	25.8	20.5
Stamen (n=1)	0.1	30.5	0.0	0.0	0.4	1.0	0.0	0.0	0.0	1.1	9.3	1.5	13.4	4.1
Ovule (n=1)	0.8	135.2	0.0	0.0	18.3	20.6	0.0	0.0	0.0	19.5	25.4	9.1	72.7	17.5
Pistil (n=3)	7.1	11.9	0.0	0.0	4.6	2.8	0.0	0.0	0.0	6.7	12.2	7.0	26.4	3.7
Sepal (n=1)	0.0	2.0	0.0	0.0	7.8	5.9	3.1	0.0	0.0	19.6	6.9	0.0	5.9	0.0
Petal (n=2)	217.4	29.2	0.0	0.0	4.2	1.2	0.8	0.6	0.0	3.7	37.3	232.9	7.4	0.0
Silique 10-20DAF (n=13)	9.4	22.3	0.0	0.0	10.3	6.0	0.0	0.0	0.0	12.8	29.1	12.1	13.7	7.7
Silique 25DAF (n=6)	6.4	20.2	0.0	0.0	11.1	11.3	0.0	0.0	0.0	18.1	42.6	3.3	12.5	23.8
Silique 30DAF (n=6)	10.7	33.8	0.0	0.0	13.7	13.1	0.0	0.0	0.0	18.7	44.7	6.1	11.0	17.6
Silique 40DAF (n=2)	6.5	22.2	0.0	0.0	7.3	8.1	0.0	0.0	0.0	10.2	19.5	11.9	2.9	6.4
Seed_35DAF_E617* (n=3)	54.9	87.7	0.0	0.0	9.6	11.7	0.0	0.0	0.0	18.1	19.4	31.2	6.9	6.6
Seed_35DAF_S14* (n=3)	0.7	217.0	0.0	0.0	18.6	15.2	0.0	0.0	0.0	18.2	34.1	54.2	6.5	12.6
Seed coat 14DAF (n=7)	0.9	2.5	0.0	0.0	7.7	4.9	0.0	0.0	0.0	5.4	1.5	0.0	3.7	1.5
Seed coat 21DAF (n=6)	38.6	69.5	0.0	0.0	19.5	14.1	0.0	0.0	0.0	16.8	24.7	0.0	34.1	12.2
Seed coat 28DAF (n=6)	120.7	151.6	0.0	0.1	19.2	14.0	0.0	0.0	0.0	20.9	42.5	0.0	95.5	42.2
Seed coat 35DAF (n=6)	92.4	90.8	0.0	0.1	18.3	16.2	0.0	0.0	0.0	18.0	48.4	0.0	91.0	42.0
Seed coat 42DAF (n=6)	111.9	82.6	0.0	0.0	14.6	13.2	0.0	0.0	0.0	21.4	245.5	0.0	63.1	27.8
Embryo (n=6)	13.5	20.4	0.0	0.0	10.5	9.8	0.0	0.0	0.0	25.4	9.5	3.2	2.1	10.9
Endosperm (n=8)	15.2	33.4	0.0	0.0	4.7	4.1	0.0	0.0	0.0	5.5	3.1	3.0	10.4	1.3
Seedling (n=9)	0.2	7.5	0.0	0.1	13.2	10.5	0.0	0.0	0.0	23.6	15.5	10.9	18.6	0.0
Stem (n=19)	126.9	227.9	0.0	0.0	29.0	25.1	0.0	0.0	0.0	67.9	131.6	59.7	117.5	19.9
Shoot (n=2)	2.1	8.2	0.0	0.0	1.8	0.9	0.0	0.0	0.0	3.9	4.9	2.3	5.4	6.0
Shoot apex (n=2)	11.7	8.1	0.0	0.0	4.6	1.6	0.0	0.0	0.0	6.4	4.3	1.1	3.4	1.3
Root seedling (n=13)	8.4	7.6	0.0	0.5	18.4	14.1	0.0	0.0	0.0	56.7	44.3	25.8	31.2	3.6

Root 30DAP (n=20)	6.5	7.4	0.0	0.6	66.3	55.6	0.0	0.0	0.0	82.5	92.6	21.6	77.8	54.7
Root 60DAP (n=2)	0.5	13.8	0.0	0.2	132.0	148.0	8.0	0.0	0.0	252.5	290.0	17.5	364.5	1.0

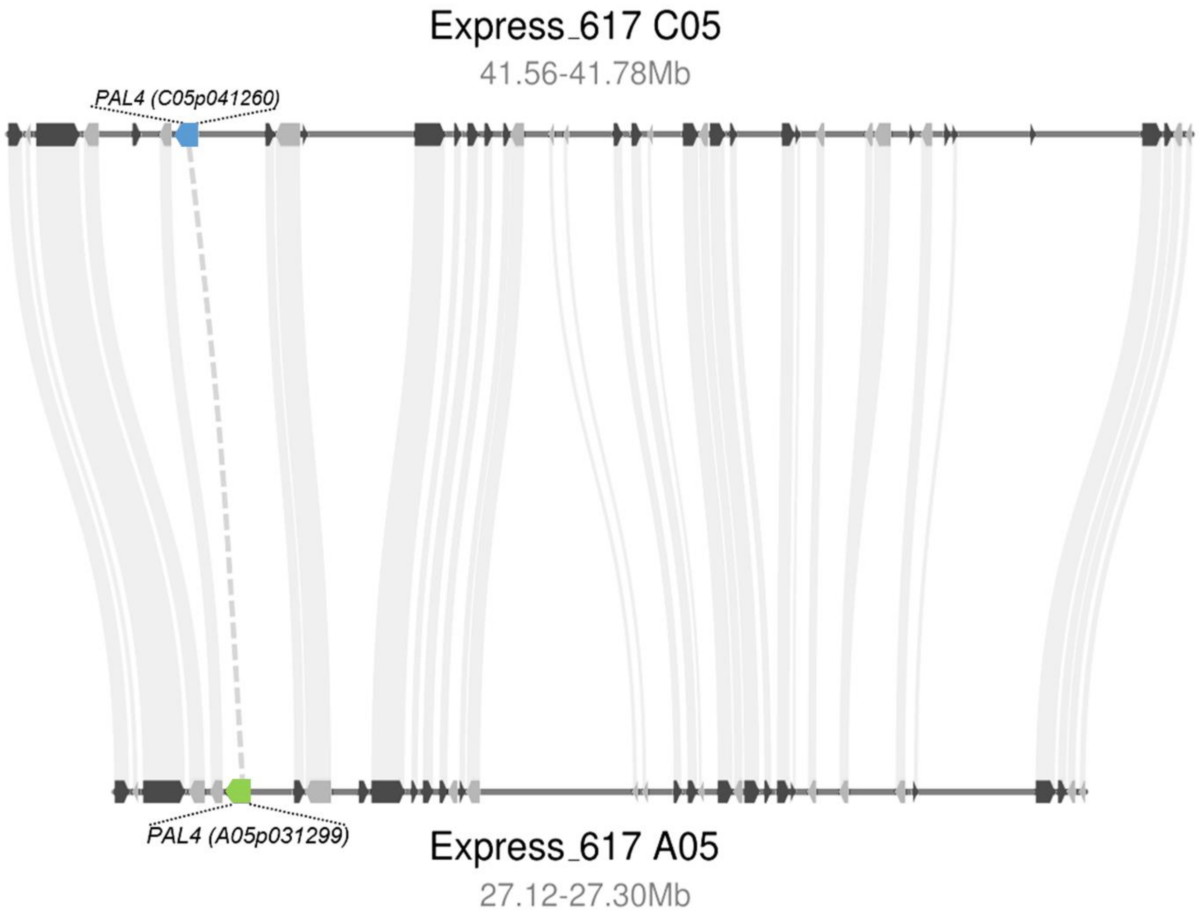

Supplementary Figure 5: *B. napus* Express 617 carries a previously not annotated A05 *PAL4* homolog and shows high local synteny between the homologous A05 region to the C05 region. The synteny plot was constructed based on the Express 617 assembly and covers the C05 region (41,56-41,78 million base pairs (Mb)) located inside the major low lignin QTL, as well as the corresponding homologous A05 region ranging from 27,12-27,30 million base pairs (Mb). Genes located on the forward strand are marked in black, while genes located on the reverse strand are marked in grey. The *PAL4* copy on C05 is highlighted in blue, while the manually annotated *PAL4* copy on A05 is marked in green. Respective novel syntenic genes are marked by a dashed line.

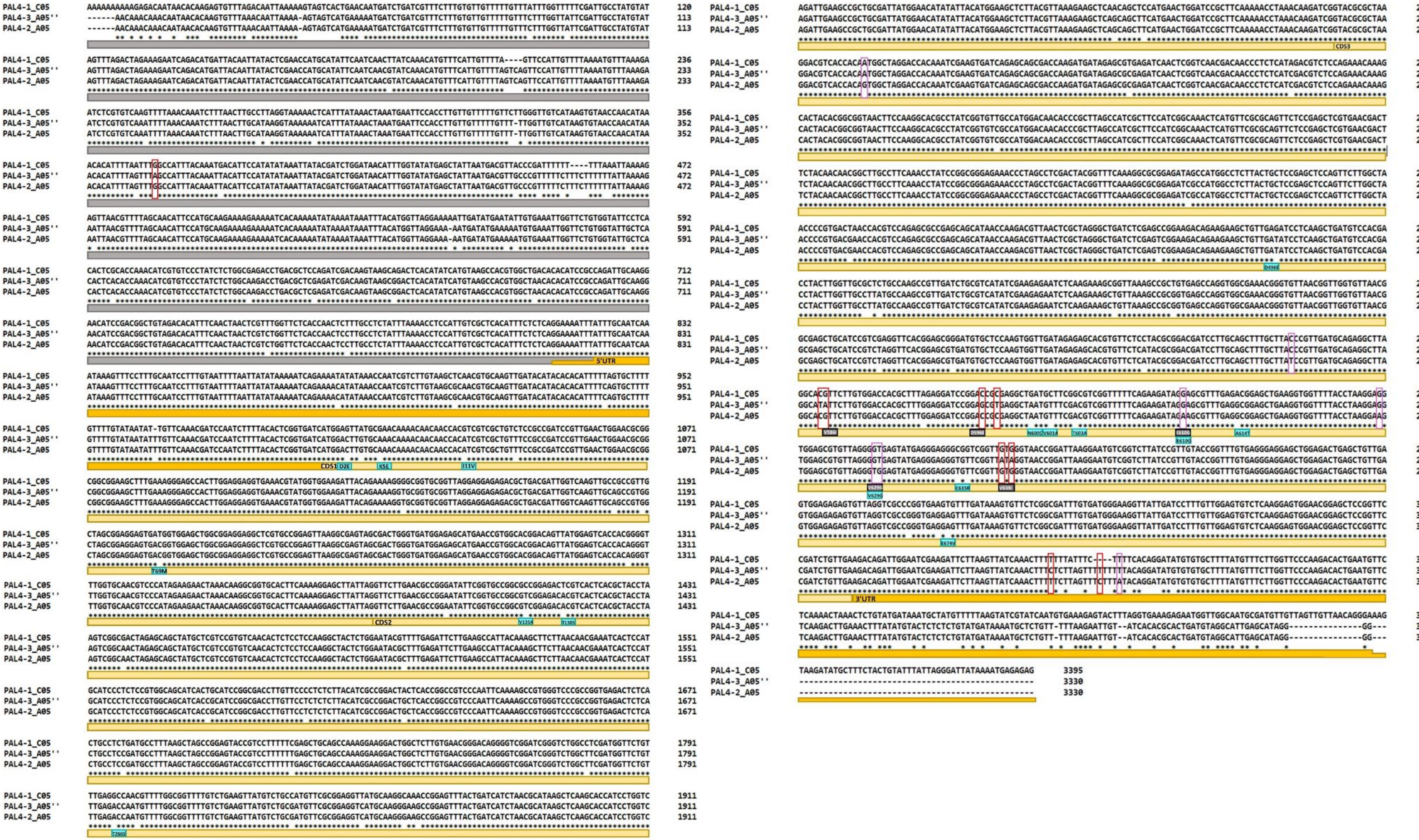
PAL4-1_C05
PAL4-3_A05''
PAL4-2_A05
5'UTR
CDS1
CDS2
CDS3
3'UTR

Supplementary Figure 6: The CDS with UTRs and upstream sequences of the A05, A05'', and C05 *PAL4* homologs. The CDS (light yellow box), UTRs (orange boxes) and 5'UTR upstream sequences (grey boxes) of the A05, A05'' and C05 *PAL4* homologs are shown. The A05'' *PAL4* sequence is identical to the A05 *PAL4* sequence except for the positions marked with red and purple rectangles. Subgenome-specific differences between A05 and A05'' *PAL4*- to the C05 *PAL4* sequence can thus be inferred by all remaining positions lacking an asterisk underneath the alignment. Positions marked with a red rectangle stress A05'' specific SNPs, while purple rectangles mark SNPs, where the A05'' PAL4 homolog carries the C05 *PAL4* allele. If one one these SNPs results in an amino acid exchange in the A05'' PAL4 sequence compared to the A05 PAL4 sequence a black box underneath each codon show the respective exchange, e.g. at position 586 a valine was exchanged by isoleucine in the A05'' amino acid sequence. Amino acid exchanged derived from subgenomes-specific SNPs are shown in cyan rectangles, e.g. at position 2, the aspartic acid (D) of A05 PAL4 is changed to glutamic acid (E) in the C05 PAL4. Clustal Omega (Sievers et al. 2011) was applied for the construction of the sequence alignment.
