# Supplementary material for: Homoeologous non-reciprocal translocation explains a major QTL for seed lignin content in oilseed rape (Brassica napus L.)
Source: Theor Appl Genet. 2023 Jul 13;136(8):172. doi: 10.1007/s00122-023-04407-w (PMC10345078; doi:10.1007/s00122-023-04407-w)
Supplement: Supplementary file 8 — Supplementary file8 (PDF 165 kb) [file 122_2023_4407_MOESM8_ESM.pdf]

Homoeologous non-reciprocal translocation explains a major QTL for seed lignin content in oilseed rape (*Brassica napus* L.)

Hanna Marie Schilbert[1,2,†], Karin Holzenkamp[3,†], Prisca Viehöver[1], Daniela Holtgräwe[1], Christian Möllers[3]

[1] Genetics and Genomics of Plants, CeBiTec and Faculty of Biology, Bielefeld University, Bielefeld, Germany
[2] Graduate School DILS, Bielefeld Institute for Bioinformatics Infrastructure (BIBI), Faculty of Technology, Bielefeld University, Germany
[3] Department of Crop Sciences, Division of Crop Plant Genetics, Georg-August-University, Göttingen, Germany
† These authors contributed equally to this work.

Corresponding author: Hanna Marie Schilbert, hschilbe@cebitec.uni-bielefeld.de

Theoretical and Applied Genetics

```
                                                                            P2            P1
Express617_C05_RB      GCTTAATCTGAAACACGGGCTTGGGTATCAAGTTTACCCTAACGGAGACGTCTTAGAAGG      60
Express617_A05_RB      GCTGAATCTCAAACACGGGCTTGGGTACCAAGTTTACCCTAACGGTGACGTCTTCGAAGG      60
SGDH14_C05_RB          GCTGAATCTCAAACACGGGCTTGGGTACCAAGTTTACCCTAACGGTGACGTCTTCGAAGG      60
SGDH14_A05_RB          GCTGAATCTCAAACACGGGCTTGGGTACCAAGTTTACCCTAACGGTGACGTCTTCGAAGG      60
                       *** ***** ***************** ***************** ******** *****

                                        R1
Express617_C05_RB      CTCTTGGATTCAGGGTTGGGGAGAAGGGCCAGGGAAGTACACTTGGGGTAACGGGAACAT      120
Express617_A05_RB      CTCTTGGATTCAGGGTTTGGGAGAAGGGCCAGGGAAGTACACGTGGGGGAACGGGAACAT      120
SGDH14_C05_RB          CTCTTGGATTCAGGGTTGGGGAGAAGGGCCAGGGAAGTACACTTGGGGTAACGGGAACAT      120
SGDH14_A05_RB          CTCTTGGATTCAGGGTTTGGGAGAAGGGCCAGGGAAGTACACGTGGGGGAACGGGAACAT      120
                       ***************** ************************ ***** ***********

Express617_C05_RB      CTATCTTGGGGATATGAAAGGTGGGAA      147
Express617_A05_RB      CTATCTTGGGGATATGAAAGGTGGGAA      147
SGDH14_C05_RB          CTATCTTGGGGATATGAAAGGTGGGAA      147
SGDH14_A05_RB          CTATCTTGGGGATATGAAAGGTGGGAA      147
                       ***************************
```

<G> 3‘ Anchor R1 reverse primer

[A/C 3‘ SNP P1 forward primer; alternative [A/T] 3‘ SNP P2 forward primer

GAAG-GGTT 22 bp non subgenome-specific region

Supplementary Figure 7: Anchored KASP assay design for homoeologous non-reciprocal translocation in SGDH14. An anchored KASP INDEL marker was designed to detect the A05/C05 insertion using the marked mismatches in both sequences on the right border.

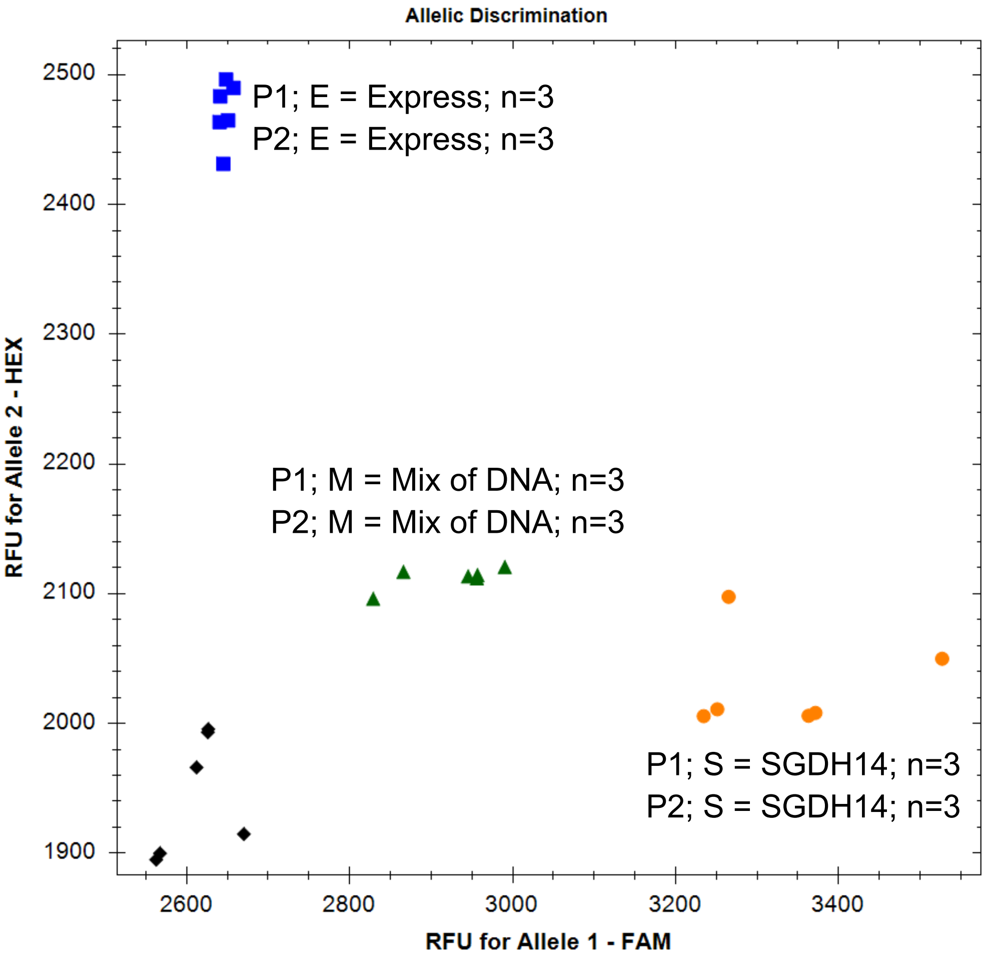

Well	Sample	Call	Type	RFU1	RFU2
A01	P1,S	Allele 1	Auto	3234	2006
A02	P1,S	Allele 1	Auto	3265	2098
A03	P1,S	Allele 1	Auto	3251	2011
A04	P1,E	Allele 2	Auto	2645	2431
A05	P1,E	Allele 2	Auto	2651	2465
A06	P1,E	Allele 2	Auto	2640	2464
A07	P1,M	Heterozygote	Auto	2866	2117
A08	P1,M	Heterozygote	Auto	2957	2114
A09	P1,M	Heterozygote	Auto	2829	2096
A10	Wasser	No Call	Auto	2612	1966
A11	Wasser	No Call	Auto	2567	1900
A12	Wasser	No Call	Auto	2670	1915
B01	P2,S	Allele 1	Auto	3363	2006
B02	P2,S	Allele 1	Auto	3372	2008
B03	P2,S	Allele 1	Auto	3527	2050
B04	P2,E	Allele 2	Auto	2641	2483
B05	P2,E	Allele 2	Auto	2658	2490
B06	P2,E	Allele 2	Auto	2648	2496
B07	P2,M	Heterozygote	Auto	2990	2121
B08	P2,M	Heterozygote	Auto	2956	2112
B09	P2,M	Heterozygote	Auto	2945	2113
B10	Wasser	No Call	Auto	2626	1993
B11	Wasser	No Call	Auto	2627	1996
B12	Wasser	No Call	Auto	2563	1895

Supplementary Figure 8: Anchored KASP assay results for homoeologous non-reciprocal translocation in SGDH14. KASP anchored SNP result of Express 617 (blue rectangles), SGDH14 (orange dots), and a mixed samples of both genotypes (green triangles) were used for KASP assay. The black diamonds mark the water controls. Two forward primers were tested (P1 and P2), which performed equally well. RFU, relative fluorescence units.
